# A simple and easy to perform synthetic route to functionalized thienyl bicyclo[3.2.1]octadienes

**DOI:** 10.3762/bjoc.16.96

**Published:** 2020-05-22

**Authors:** Dragana Vuk, Irena Škorić, Valentina Milašinović, Krešimir Molčanov, Željko Marinić

**Affiliations:** 1 Department of Organic Chemistry, Faculty of Chemical Engineering and Technology, University of Zagreb, Marulićev trg 19, 10000 Zagreb, Croatia; 2 Division of Physical Chemistry, Rudjer Bošković Institute, Bijenička cesta 54, 10000 Zagreb, Croatia; 3 NMR Centre, Ruđer Bošković Institute, Bijenička cesta 54, 10000 Zagreb, Croatia

**Keywords:** bicyclo[3.2.1]octadiene, photocyclization, thiophene, Vilsmeier–Haack reaction, Wittig reaction

## Abstract

In order to prepare novel polycyclic derivatives of bicyclo[3.2.1]octadiene systems fused with a thiophene ring, photochemical cyclization and aldol condensation reactions were carried out. The starting substrates were easily obtained by a Vilsmeier–Haack reaction of bicyclo[3.2.1]octadiene thiophene derivatives with dimethylformamide. From the obtained carbaldehydes, novel methyl, methoxy, and cyano-substituted styryl thienobenzobicyclo[3.2.1]octadiene derivatives were synthesized through Wittig reactions and subjected to photochemical cyclization, in terms of obtaining the new annulated structures. As part of this study, the aldol reaction of the starting 2-substituted carbaldehyde with acetone was also performed, which produced the thieno-fused benzobicyclo[3.2.1]octadiene compound with an extended conjugation.

## Introduction

**Figure 1:**
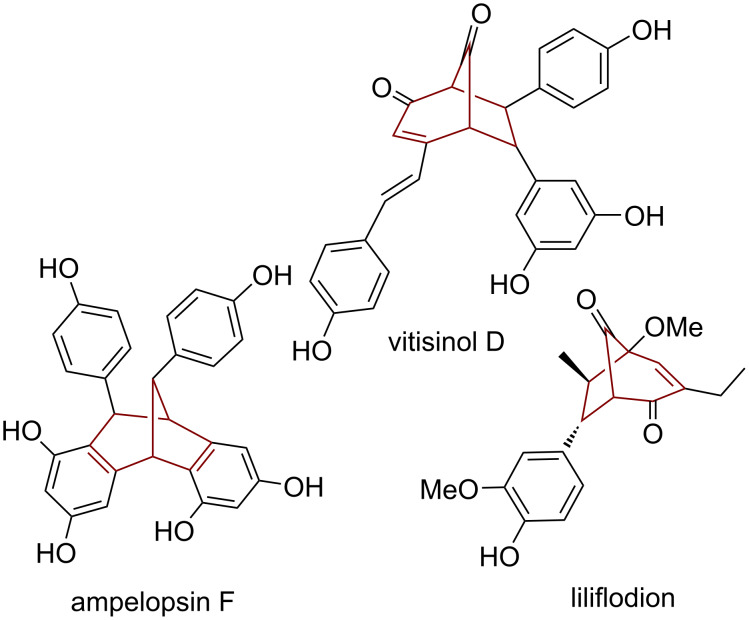
Known biologically active bicyclo[3.2.1]octenes/octadienes.

**Figure 2:**
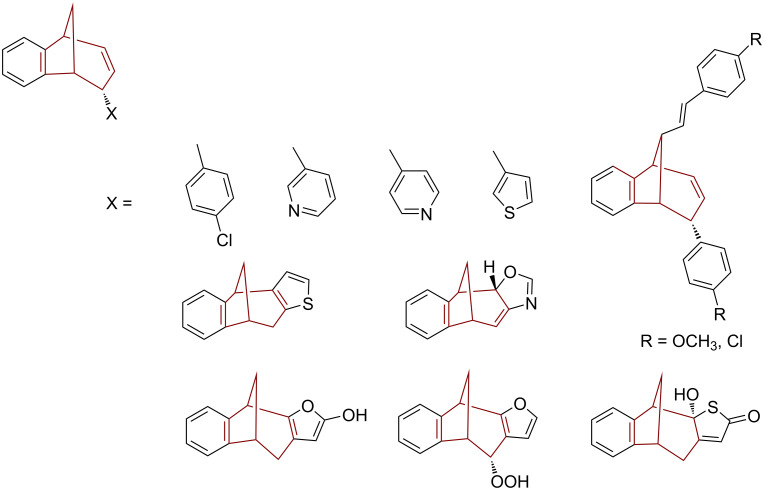
Previously prepared bicyclo[3.2.1]octenes/octadienes with cholinesterase inhibitory properties.

The bicyclo[3.2.1]octane skeleton has become the subject of intensive research in recent years [1–3]. Its presence in numerous biologically active natural compounds (Figure 1) [4–7], their strenuous isolation procedures from plants, as well as their complicated multistage synthesis due to the complexity of their structure, encouraged us to develop a simple one-step synthetic procedure based on a photochemical methodology [8–21]. By using a simple photochemical procedure, it was possible to obtain a whole library of novel bicyclo[3.2.1]octadiene derivatives, available for further functionalization, which could enable the easier investigation of the relationship between structure and biological activity. During our previous investigation a series of functionalized compounds with a benzobicyclo[3.2.1]octadiene skeleton was prepared, among which some showed cholinesterase inhibitory properties (Figure 2) [2–3].

**Scheme 1:**
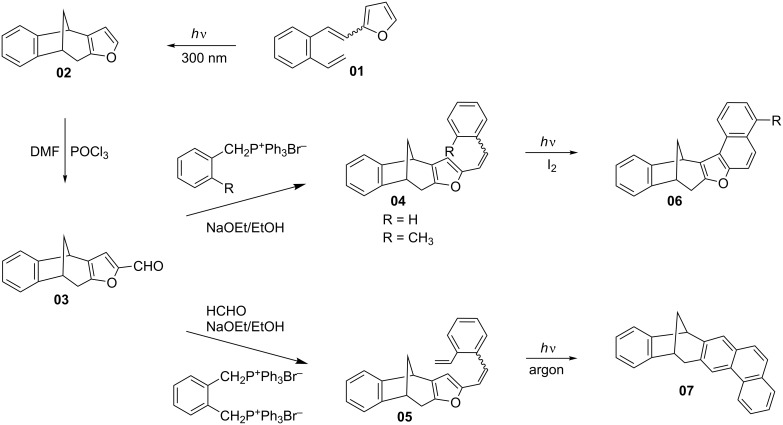
Synthesis of annulated furobenzobicyclo[3.2.1]octadiene compounds.

The aim of this study was to prepare novel thiophene bicyclo[3.2.1]octadiene derivatives with a structure convenient for the introduction of new functional groups. Further on, the study aimed at expanding the compound library and at creating preconditions for further biological investigations. This work represents a rational continuation of the research [17], previously done on similar furobicyclo[3.2.1]octadiene compounds. The previous study included the synthesis of aldehyde **03**, which was via the corresponding styryl derivatives converted to the annulated products **04**–**07**. These compounds were of particular importance due to their rigid methano-bridged junction of two aromatic units (Scheme 1). The idea herein was to prepare thienyl analogues of the annulated furyl derivatives, as substrates suitable for biological testing and/or new precursors for further functionalization.

## Results and Discussion

**Scheme 2:**
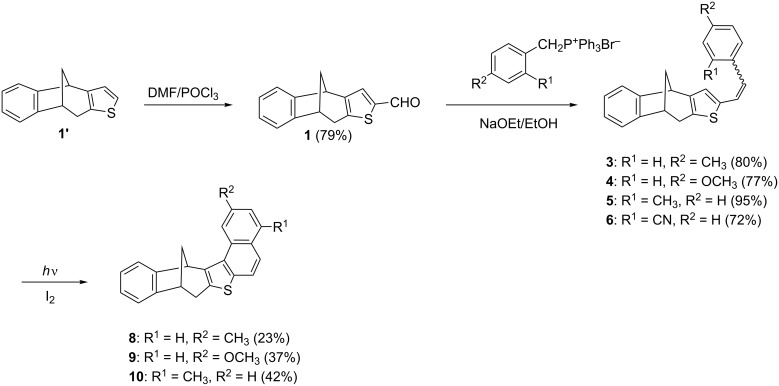
Synthesis of annulated thiophenebicyclo[3.2.1]octadiene compounds **8**-**10**.

**Scheme 3:**
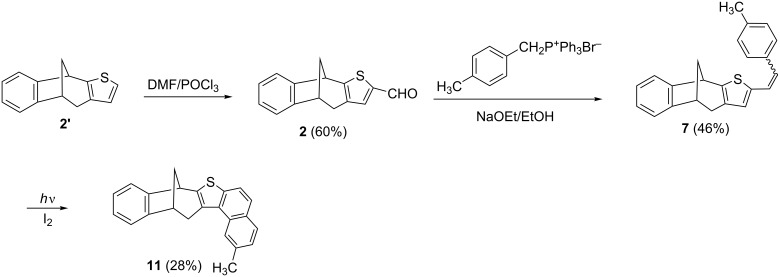
Synthesis of compound **11**.

As starting precursors two bicyclic thiophene derivatives **1'** and **2'**, with different position of the sulfur in thiophene moiety, were selected. The compounds **1'** and **2'** were prepared according to the previously reported one-step photochemical methodology [15], and subjected to the Vilsmeier–Haack reaction (Scheme 2, Scheme 3), respectively. After chromatographic purification, the aldehydes **1** and **2** were obtained in very good yields (**1**: 79%; **2**: 68%), and subsequently used as novel starting substrates for further addition/condensation reactions.

**Figure 3:**
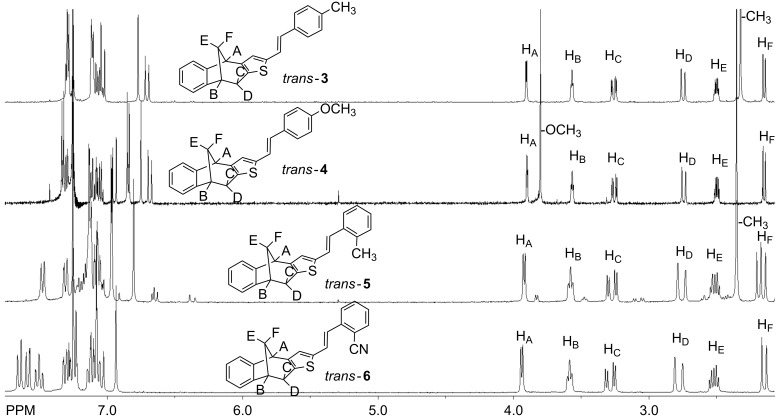
^1^H NMR spectra (CDCl_3_) for the *trans*-isomers **3**–**6**.

The Wittig reaction of the prepared aldehydes with the corresponding triphenylphosphonium salts provided five new styryl derivatives **3**–**7** as mixtures of *cis* and *trans*-isomers. The isomers of compounds **3** and **4** were separated by column chromatography and completely spectroscopically characterized, while in the case of compounds **5**–**7**, only *trans*-isomers were obtained. Figure 3 presents parts of the ^1^H NMR spectra of the *trans*-isomers **3**–**6** as representative examples. The detailed analysis of all new compounds' NMR spectra can be found in Supporting Information File 1. The ^1^H NMR spectra of the presented examples confirmed the conservation of the bicyclic core. Six proton pattern, characteristic for these bicyclic systems, were clearly visible, with similar shifts in all cases, due to the only slight impact of the substituents on the phenyl moiety. The most significant difference was related to the protons of the methoxy group, which were shifted upfield as expected. Also a slight impact of *para*-substituents on the chemical shifts of the aromatic protons could be observed, with the proton chemical shift shifting upfield.

**Figure 4:**
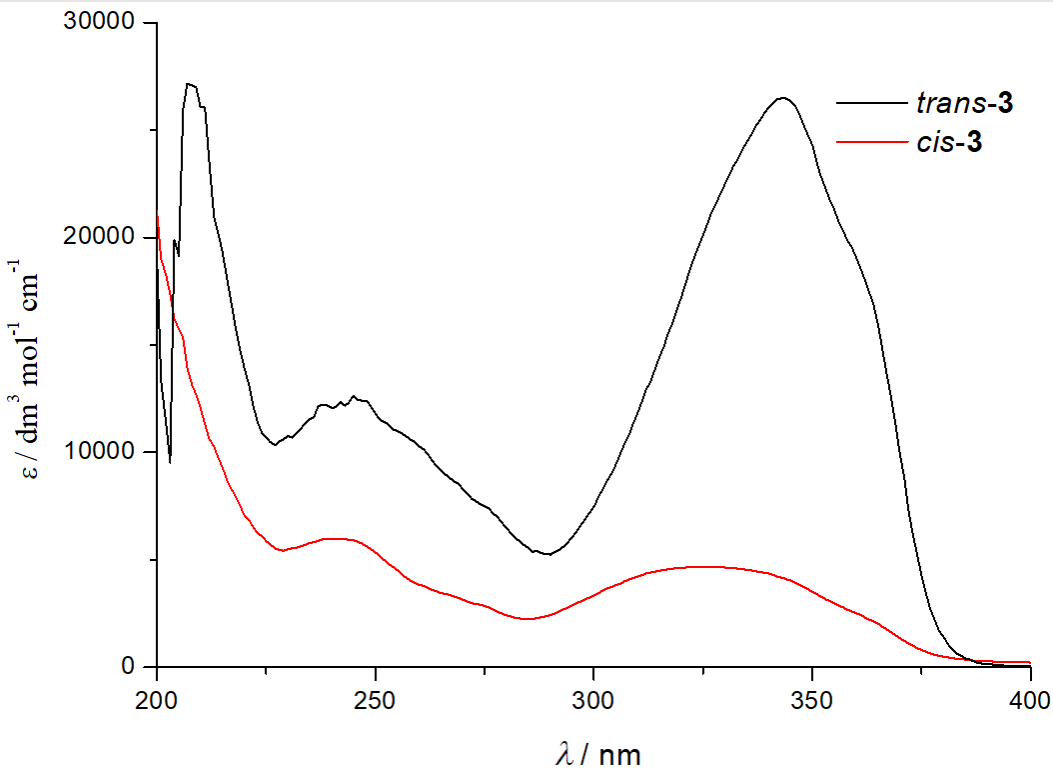
UV spectra in ethanol (95%) of the *cis*- and *trans*-isomers of compound **3**.

The comparison of the UV spectra of the *cis*- and *trans*-isomers of compound **3** (Figure 4) showed the expected bathochromic and hyperchromic shifts of the *trans*-isomers, due to the planarity of the structure.

**Figure 5:**
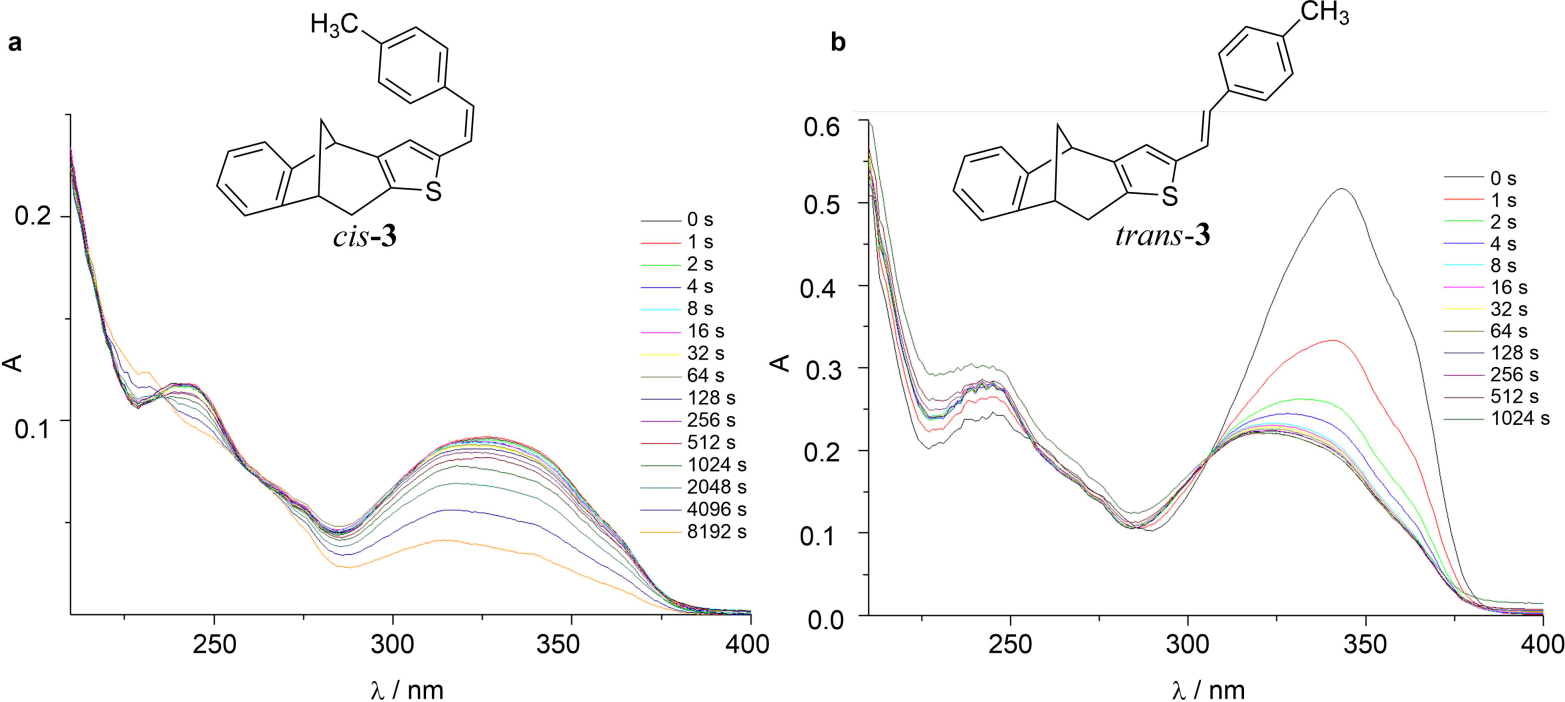
Photolysis spectra of *cis*-**3** (a) and *trans*-**3** (b) in ethanol (95%).

Further, the separated isomers of **3**–**7** were irradiated and the reaction course followed by UV spectroscopy. In all cases, the longest wavelength absorption band gradually disappeared upon irradiation. Based on previous research, it was assumed, that the preliminary process could be a photoisomerization, which could be accompanied by a photochemical annulation. The photolysis spectra of compound's **3** isomers are shown in Figure 5, as representative examples.

**Figure 6:**
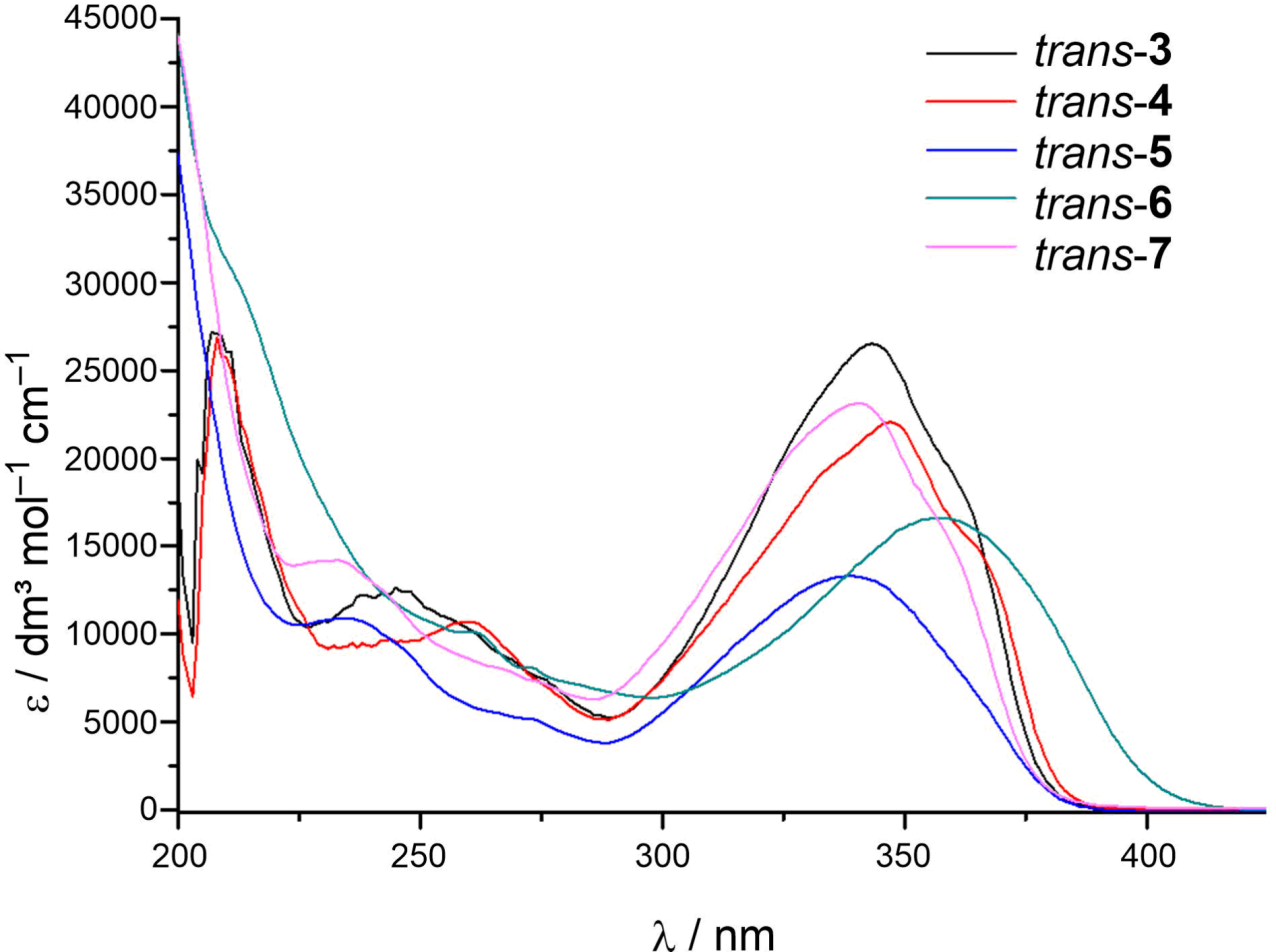
UV spectra in ethanol (95%) of the *trans*-isomers of compounds **3**–**7**.

Figure 6 presents the UV spectra of products' **3**–**7** *trans*-isomers. All the isomers showed an absorption maxima between 300–400 nm. It can be noticed that *p-*substituents (*trans*-**3**, *trans*-**4**, and *trans*-**7**) enabled a higher value of molar extinction coefficients, in comparison to *o*-substituted compounds (*trans*-**5** and *trans*-**6**). The cyano-substituted compound *trans*-**6** showed the largest bathochromic shift, in regards to the methyl and methoxy-substituted compounds (*trans*-**3**–**5**, *trans*-**7**).

**Figure 7:**
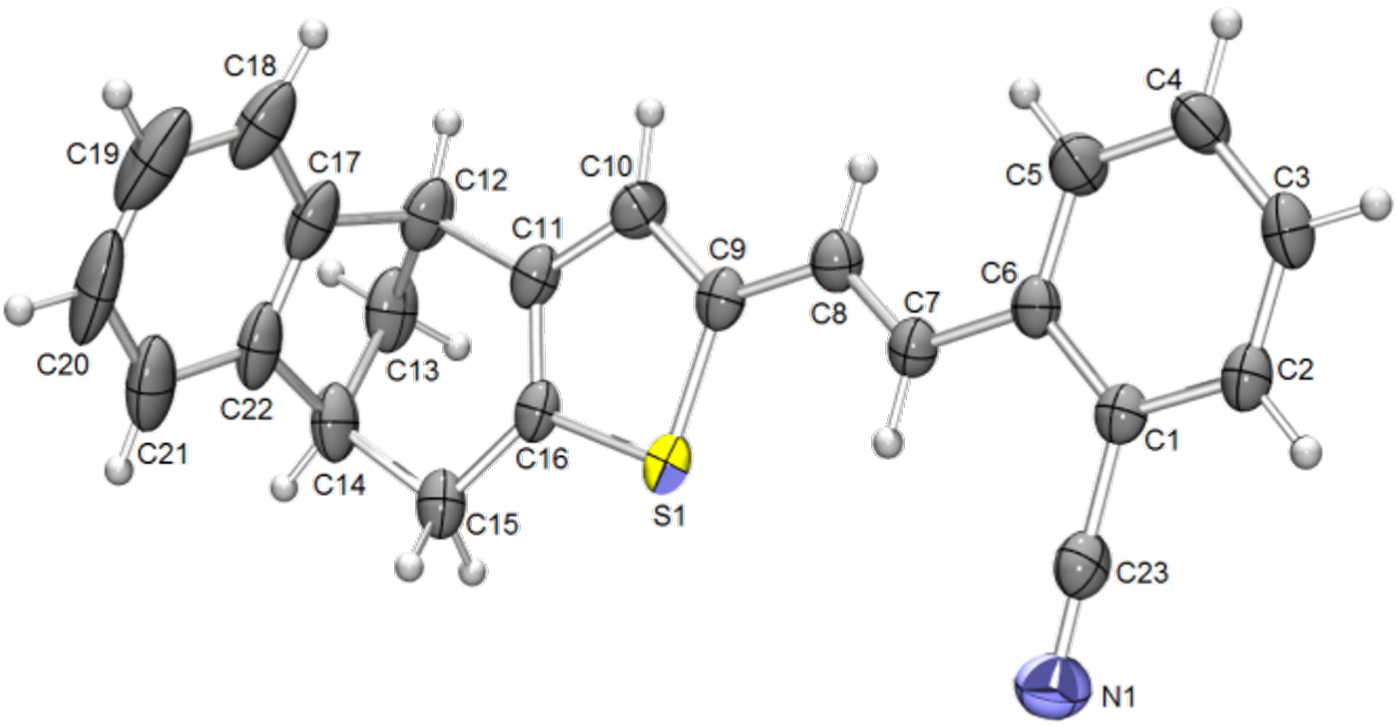
Molecular structure of compound *trans*-**6**. Displacement ellipsoids are drawn for the probability of 30% and hydrogen atoms are shown as spheres of arbitrary radii.

**Figure 8:**
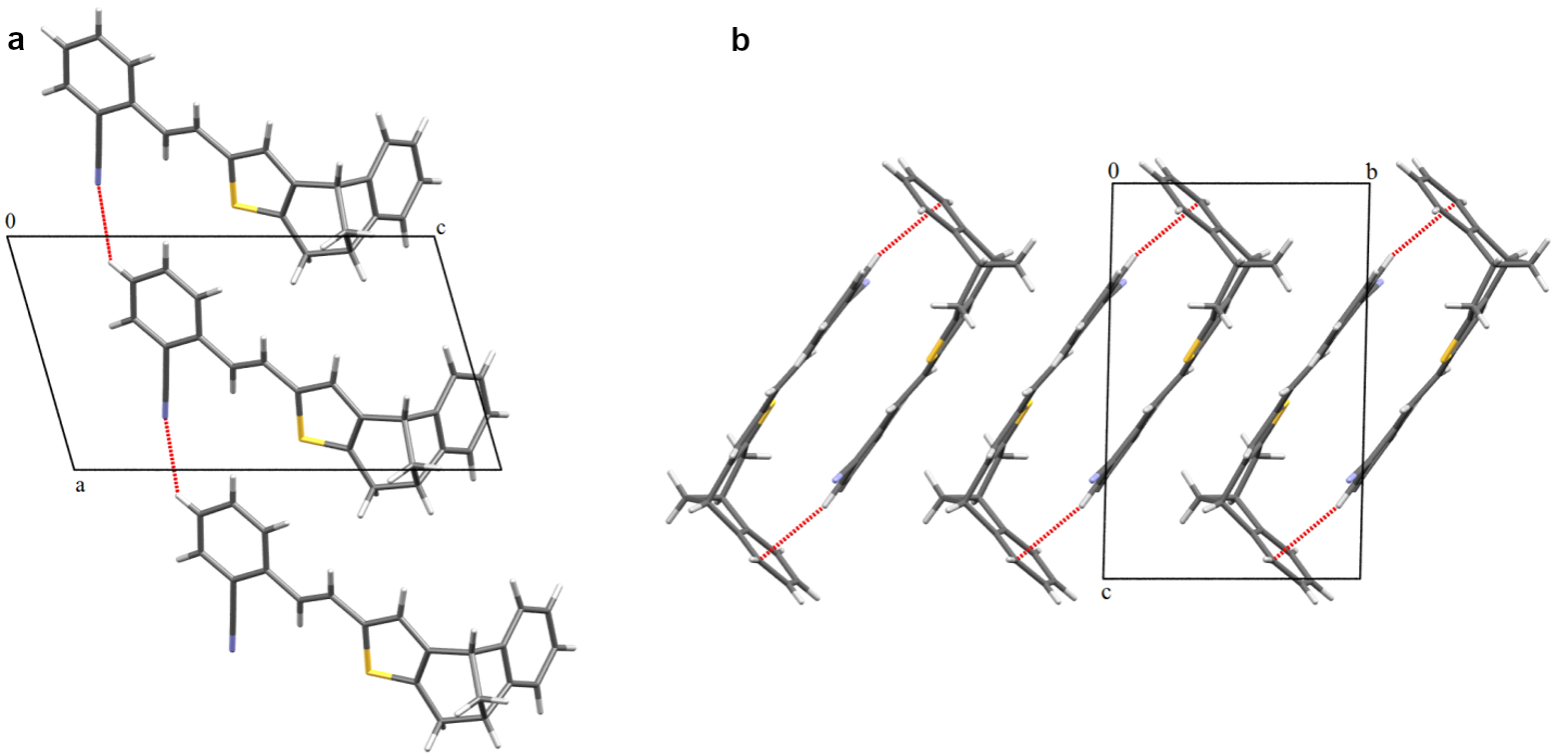
Crystal packing of *trans*-**6**. (a) Chain parallel to [100] and (b) chain parallel to [010].

The structure of *trans*-**6** was also confirmed by X-ray analysis (Figure 7). The compound crystallized in the 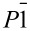 space group, with the molecular symmetry *C*_i_. The crystal packing is presented in Figure 8.

The next synthesis step involved the preparation of the annulated bicyclo[3.2.1]octadiene derivatives by irradiating the toluene solution of compound's **3**–**7** mixture of *cis*- and *trans*-isomers in the presence of iodine (Scheme 2 and Scheme 3). The electrocyclization reactions were successfully implemented in most cases and photoproducts **8**–**11** were obtained in moderate yields. The only exception was the cyano derivative **6** which was proven to be non-reactive, since the reaction mixture showed solely the presence of the initial *cis*- and *trans*-isomers.

**Figure 9:**
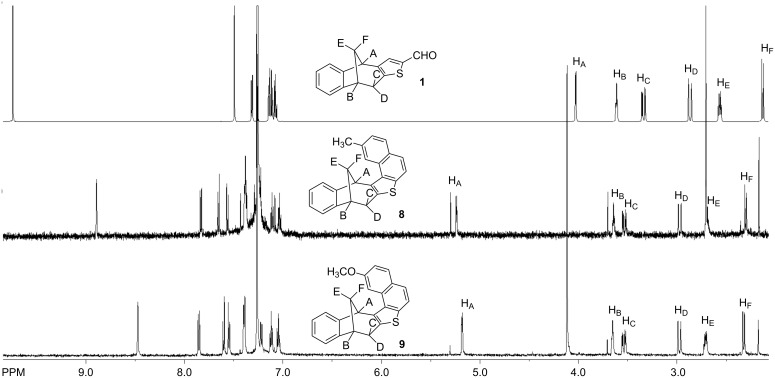
^1^H NMR spectra (CDCl_3_) of compounds **1**, **8**, and **9**.

Figure 9 presents the ^1^H NMR spectra of photoproducts **8** and **9**, in comparison to the spectra of the starting aldehyde **1**. The effect of the substituent could be seen through a shift of the aromatic singlet, which is, in the case of methoxy-substituted derivative **9**, shifted downfield, due to the electronic and anisotropic effect of the methoxy group.

**Scheme 4:**
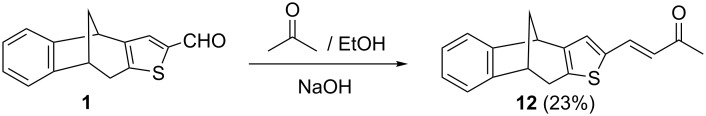
Synthesis of compound **12**.

In continuation of the study herein presented, the aldol condensation reaction of the bicyclo[3.2.1]octadiene aldehyde **1** and acetone was conducted (Scheme 4). After purification of the reaction mixture the product **12** was obtained. The aim of this experiment was to obtain a system with an extended conjugation of the heteroaromatic moiety under mild conditions, while leaving the bicyclic skeleton preserved.

**Figure 10:**
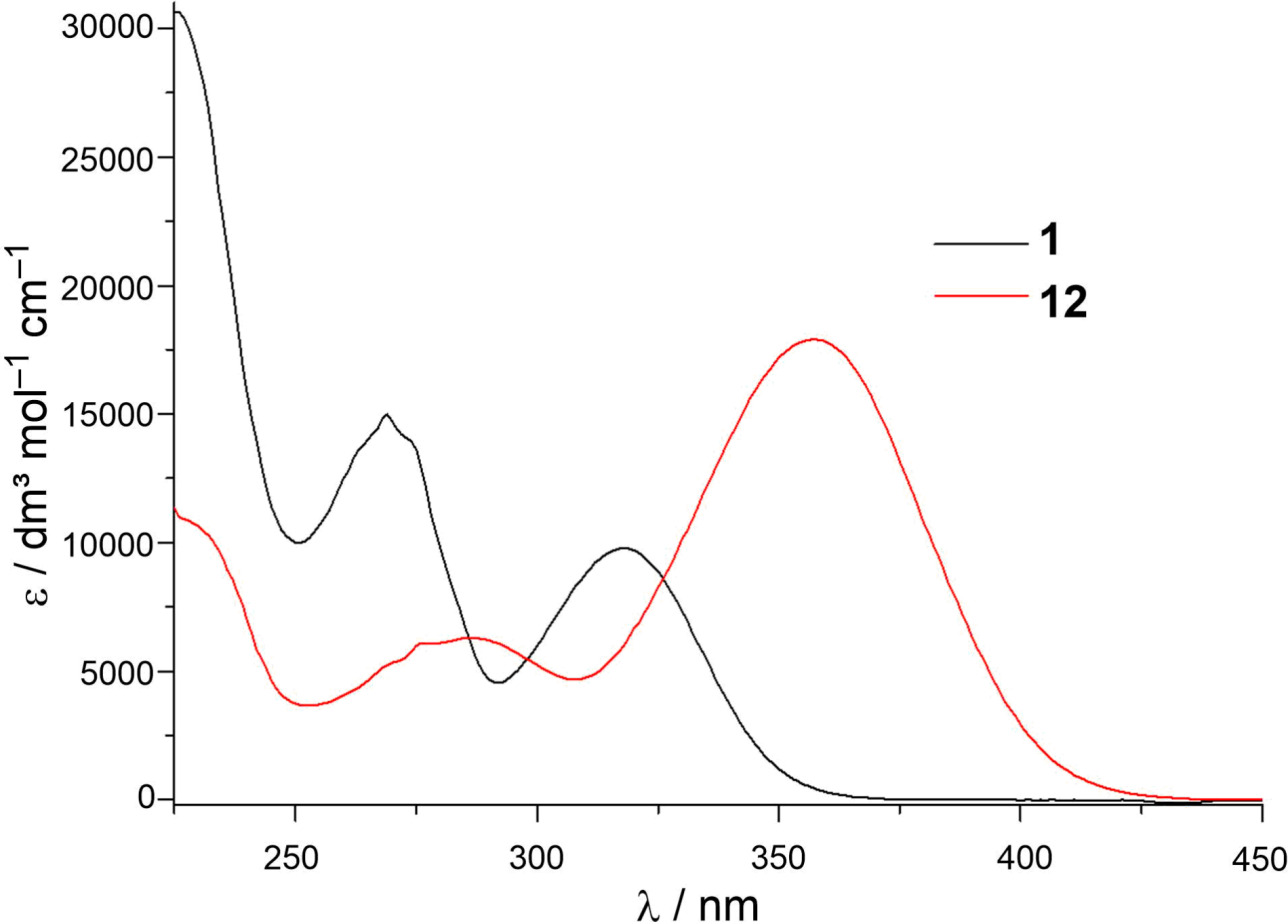
UV spectra of compounds **1** and **12** in ethanol (95%).

**Figure 11:**
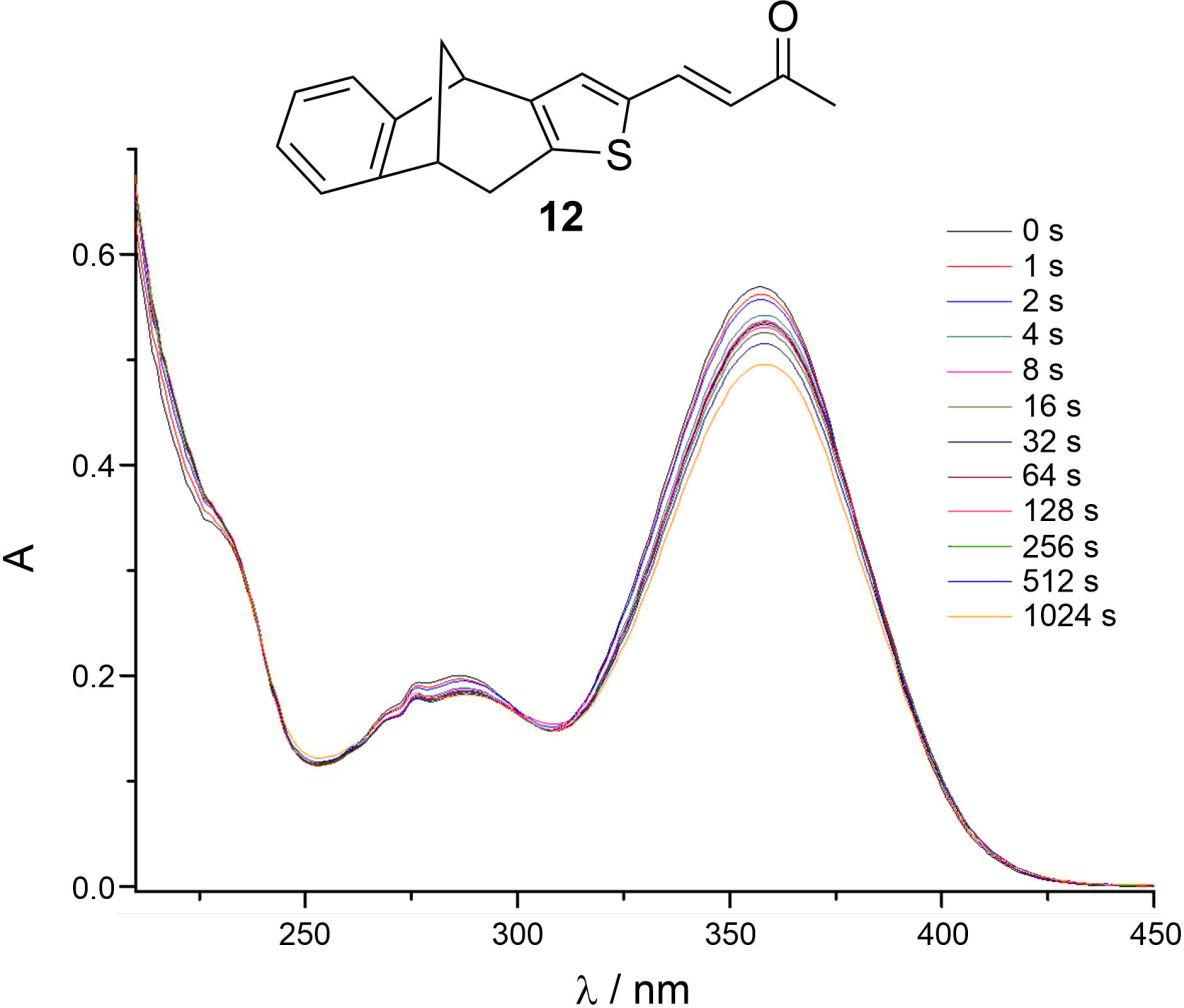
Photolysis spectra of compound **12** in ethanol (95%).

The UV spectrum of the aldol product **12**, in comparison to the starting aldehyde **1**, showed the expected red shift, under the prolonged conjugation in product **12** (Figure 10). Contrary to the results obtained on the styryl analogs **3**–**7**, the preliminary irradiation experiments of compound **12** indicated its lower photoreactivity, as it was shown by only a slight decrease of the absorption band (Figure 11).

**Scheme 5:**
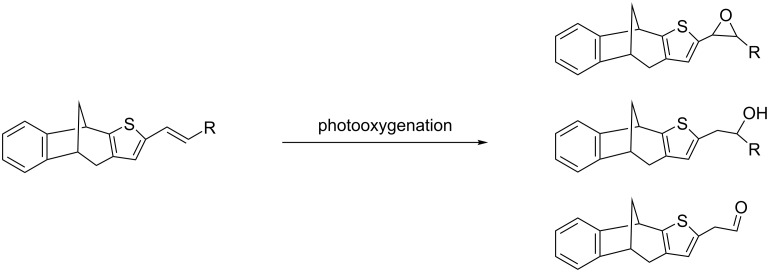
Possible outcomes of future photocatalytic oxygenation reactions of new benzobicyclo[3.2.1.]octadienes.

As previously emphasized, the prepared products **3**–**7** and **12**, due to the presence of a double bond in their structure, could serve as potential starting precursors for further functionalization. These functionalizations, beside the addition reaction, could involve photooxygenation reactions (Scheme 5) [1,22–25], previously studied in our laboratory. These reactions could result in a completely new spectrum of products, with preserved bicyclo[3.2.1.]octadiene skeleton, crucial for biological testing.

## Conclusion

From the two starting thiophene derivatives **1** and **2**, ten novel products **3**–**12** have been prepared by a simple and low-cost procedure, paving the way to new researches, some of which could be directed toward inclusion of new heterocycles. Due to their indicative structure the prepared compounds **1**–**12** are candidates for biological assays. The novel styryl derivatives **3**–**7** and **12** could also find application in further research for functionalization of the bicyclo[3.2.1.]octadiene core.

## Supporting Information

File 1 Experimental details, copies of spectra and X-ray crystallographic data.
