# Supplementary material for: A simple and easy to perform synthetic route to functionalized thienyl bicyclo[3.2.1]octadienes
Source: Beilstein J Org Chem. 2020 May 22;16:1092–9. doi: 10.3762/bjoc.16.96 (PMC7277534; doi:10.3762/bjoc.16.96)
Supplement: File 1 — Experimental details, copies of spectra and X-ray crystallographic data. [file Beilstein_J_Org_Chem-16-1092-s001.pdf]

BEILSTEIN JOURNAL OF ORGANIC CHEMISTRY

Supporting Information

for

A simple and easy to perform synthetic route to functionalized thienyl bicyclo[3.2.1]octadienes

Dragana Vuk, Irena Škorić, Valentina Milašinović, Krešimir Molčanov and Željko Marinić

Beilstein J. Org. Chem. **2020,** *16,* 1092–1099. doi:10.3762/bjoc.16.96

Experimental details, copies of spectra and X-ray crystallographic data

License and Terms: This is a supporting information file under the terms of the Creative Commons Attribution License (http://creativecommons.org/licenses/by/4.0). Please note that the reuse, redistribution and reproduction in particular requires that the authors and source are credited.
The license is subject to the *Beilstein Journal of Organic Chemistry* terms and conditions: (https://www.beilstein-journals.org/bjoc)

Table of contents

Experimental data for compounds **1-12** S2

X-ray Crystallography S11

Copies of spectra S12

Experimental section

General experimental information

The ^{1}H NMR spectra were recorded on a spectrometer at 300 and 600 MHz. The ^{13}C NMR spectra were registered at 75 and 150 MHz. All NMR spectra were measured in $CDCl_3$ using tetramethylsilane as reference. The assignment of the signals was based on 2D-CH correlation and 2D-HH-COSY and NOESY experiments. UV spectra were measured on an UV/VIS Cary 50 spectrophotometer. IR spectra were recorded on an FTIR-ATR (film). Irradiation experiments were performed in a quartz vessel in toluene solution in a photochemical reactor equipped with 3500 Å lamps. Melting points were obtained using microscope equipped apparatus and are uncorrected. HRMS analysis were carried out on a Agilent 6545 Q-TOF LC/MS (G6545B) connected to an Agilent 1290 Infinity II UPLC system (High pressure pump G7120A, Autosampler G7129B, Column compartment G7116B, DAD detector G7117B). Silica gel (0.063–0.2 mm) was used for chromatographic purifications. Thin-layer chromatography (TLC) was performed silica gel 60 F254 plates. Solvents were purified by distillation. Starting compounds **A** and **B** were previously synthesized in our laboratory [1].

Synthesis of starting compounds 1 and 2 (Vilsmeier–Haack formylation) according to an analogous procedure as described in [2]

To a cooled and stirred solution (12 °C) of starting bicyclic derivative **1'** or **2'** (0.009 mol) in dry *N,N*-dimethylformamide (0,8 mL, 0.010 mol) phosphorus oxychloride (1.038 mmol) was added. After 15 minutes, the reaction mixture was allowed to gradually warm to room temperature and stirred for 24 h. After quenching the reaction with 10% NaOH (2 mL), the product was extracted with diethyl ether (60 mL), washed with water and dried with $MgSO_4$. After column chromatography on silica gel using petroleum ether/diethyl ether (0–10%) as eluent, products **1** and **2** were isolated, respectively.

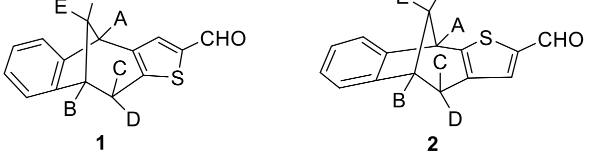

1 **2**

[1] Vidaković, D.; Škorić, I.; Horvat, M.; Marinić, Ž.; Šindler-Kulyk, M. *Tetrahedron* **2008,** *64*, 3928-3934.

[2] Kikaš, I.; Škorić, I;. Marinić, Ž.; Šindler-Kulyk, M. *Tetrahedron* **2010,** *66*, 9405-9414

5-Thiatetracyclo[6.6.1.0^{2,6}.0^{9,14}]pentadeca-2(6),3,9,11,13-pentaene-4-carbaldehyde (1): R_f 0.31 (petroleum ether/diethyl ether); colorless crystals; mp 128–130 °C; UV (96% EtOH) λ_{max}/nm (ε/dm^3 mol^{-1} cm^{-1}): 318 (9727), 269 (14811), 226 (30667, sh), 207 (72993); ^{1}H NMR ($CDCl_3$, 600 MHz) δ/ppm: 9,74 (s, 1H, CHO), 7.49 (s, 1H, H-t), 7.32 (d, 1H, J = 7.5 Hz, H-ar), 7.14 (d, 1H, J = 7.5 Hz, H-ar), 7.12 (dt, 1H, J = 7.5; 1.0 Hz, H-ar), 7.07 (dt, 1H, J = 7.5; 1.0 Hz, H-ar), 4.03 (d, 1H, $J_{A,E}$ = 4.4 Hz, H-A), 3.61 (t, 1H, $J_{B,C}$ = 4.8 Hz, H-B), 3.34 (dd, 1H, $J_{B,C}$ = 4.8; $J_{C,D}$ = 17.7 Hz, H-C), 2.87 (d, 1H, $J_{C,D}$ = 17.7 Hz, H-D), 2.54–2.59 (m, 1H, H-E), 2.13 (d, 1H, $J_{E,F}$ = 10.5 Hz, H-F); ^{13}C NMR ($CDCl_3$, 150 MHz) δ/ppm: 181.68 (d), 149.89 (s), 144.43 (s), 143.91 (s), 143.47 (s), 140.24 (s), 133.41 (d), 126.36 (d), 126.34 (d), 123.32 (d), 120.63 (d), 41.79 (d), 41.34 (t), 39.49 (d), 32.05 (t); IR ν_{max}/cm^{-1} 2939, 1467, 1294, 1150, 967, 850; HRMS: M^+_{calcd} 240.0609; M^+_{found} 240.0610.

3-Thiatetracyclo[6.6.1.0^{2,6}.0^{9,14}]pentadeca-2(6),4,9,11,13-pentaene-4-carbaldehyde (2): R_f 0.31 (petroleum ether / diethyl ether 10:1); colourless crystals; mp 75 – 77 °C; UV (96% EtOH) λ_{max}/nm (ε/dm^3 mol^{-1} cm^{-1}): 317 (9804), 265 (8881); ^{1}H NMR ($CDCl_3$, 600 MHz) δ/ppm: 9.71 (s, 1H, CHO), 7.30 – 7.33 (m, 2H, H-ar), 7.12 – 7.17 (m, 2H, H-ar), 7.09 (dt, 1H, J = 7.8; 1.3 Hz, H-ar), 4.09 (d, 1H, $J_{A,E}$ = 4.8 Hz, H-A), 3.58 (t, 1H, $J_{B,C}$ = 5.1 Hz, H-B), 3.16 (dd, 1H, $J_{B,C}$ = 5.1; $J_{C,D}$ = 17.0 Hz, H-C), 2.69 (d, 1H, $J_{C,D}$ = 17.0 Hz, H-D), 2.60–2.65 (m, 1H, H-E), 2.19 (d, 1H, $J_{E,F}$ = 10.2 Hz, H-F); ^{13}C NMR ($CDCl_3$; 150 MHz) δ/ppm: 152.28 (s), 148.41 (s), 144.41 (s), 137.79 (d), 131.96 (s), 126.79 (d), 126.38 (d), 123.25 (d), 120.95 (d), 41.88 (d), 41.19 (t), 38.90 (d), 31.43 (t); IR ν_{max}/cm^{-1} 2948, 1656, 1445, 938; HRMS: M^+_{calcd} 240.0609; M^+_{found} 240.0609.

General procedure for the synthesis of compounds 3–7 (Wittig reaction) according to an analogous procedure as described in [3]

To a stirred solution of the corresponding phosphonium salt (1.25 equiv) and the aldehyde **1** or **2** (0.5 mmol) in absolute ethanol (100 mL), a solution of sodium ethoxide (14 mg, 0.625 mmol in 5 mL of absolute ethanol) was added dropwise, respectively. The reaction was complete within 3–4 h (usually left standing overnight). After removal of the solvent, the residue was worked up with water and toluene. The toluene extracts were dried (anhydrous $MgSO_4$) and concentrated. The

[3] Vuk, D.; Potroško, D.; Šindler-Kulyk, M.; Marinić, Ž.; Molčanov, K.; Kojić-Prodić, B.; Škorić, I. *J. Mol. Struct.*, **2013,** *1051,* 1-14

crude mixture was purified and the isomers of products **3**–**7** were separated, respectively, by repeated column chromatography on silica gel using petroleum ether as the eluent.

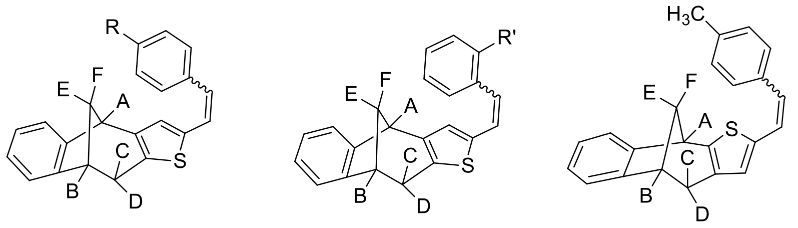

3: R = CH_3
4: R = OCH_3
5: R' = CH_3
6: R' = CN
7

4-[(*Z*)-2-(4-Methylphenyl)ethenyl]-5-thiatetracyclo[6.6.1.0^{2,6}.0^{9,14}]pentadeca-2(6),3,9(14),10,12-pentaene (*cis*-3): Yield 21%; R_f 0.77 (petroleum ether / diethyl ether 10:1); colourless oil; UV (96% EtOH) λ_{max}/nm (ε/dm^3 mol^{-1} cm^{-1}): 329 (4693), 242 (5957); ^{1}H NMR ($CDCl_3$, 600 MHz) δ/ppm: 7.23 (d, 2H, *J* = 7.6 Hz, H-ar), 7.14 (d, 2H, *J* = 7.6 Hz, H-ar), 7.03-7.12 (m, 4H, H-ar), 6.75 (s, 1H, H-t), 6.54 (d, 1H, *J* = 11.3 Hz, H-et), 6.40 (d, 1H, *J* = 11.3 Hz, H-et), 3.87 (d, 1H, $J_{A,E}$ = 3.6 Hz, H-A), 3.53 (t, 1H, $J_{B,C}$ = 5.2 Hz, H-B), 3.15 (dd, 1H, $J_{B,C}$ = 5.2; $J_{C,D}$ = 16.9 Hz, H-C), 2.62 (d, 1H, $J_{C,D}$ = 16.9 Hz, H-D), 2.45–2.55 (m, 1H, H-E), 2.38 (s, 3H, -CH_3), 2.11 (d, 1H, $J_{E,F}$ = 10.7 Hz, H-F); ^{13}C NMR ($CDCl_3$; 150 MHz) δ/ppm: 150.69 (s), 144.51 (s), 142.11 (s), 140.82 (s), 136.58 (s), 134.06 (s), 131.81 (s), 128.59 (d, d), 128.53 (d, d), 128.17 (d), 127.72 (d), 126.66 (d), 125.89 (d), 125.74 (d), 123.03 (d), 122.79 (d), 120.30 (d), 41.59 (d, C_A), 41.58 (t, $C_{E,F}$), 39.97 (d, C_B), 31.14 (t, $C_{C,D}$), 20.83 (q); IR ν_{max}/cm^{-1} 2940, 1668, 1458, 1264, 950, 821.

4-[(*E*)-2-(4-Methylphenyl)ethenyl]-5-thiatetracyclo[6.6.1.0^{2,6}.0^{9,14}]pentadeca-2(6),3,9(14),10,12-pentaene (*trans*-3): Yield 59 %, R_f 0.75 (petroleum ether / diethyl ether 10:1); colourless crystals; mp 115 – 117 °C; UV (96% EtOH) λ_{max}/nm (ε/dm^3 mol^{-1} cm^{-1}): 344 (26614), 245 (12509), 207 (27089); ^{1}H NMR ($CDCl_3$, 600 MHz) δ/ppm: 7.29 (d, 2H, *J* = 7.8 Hz, H-ar), 7.11 (d, 2H, *J* = 7.8 Hz, H-ar), 7.10-7.13 (m, 2H, H-ar), 7.08 (dt, 1H, *J* = 7.8; 1.1 Hz, H-ar), 7.05 (dt, 1H, *J* = 7.8; 1.1 Hz, H-ar), 7.03 (d, 1H, *J* = 16.2 Hz, H-et), 6.77 (s, 1H, H-t), 6.71 (d, 1H, *J* = 16.2 Hz, H-et), 3.91 (d, 1H, $J_{A,E}$ = 4.0 Hz, H-A), 3.57 (t, 1H, $J_{B,C}$ = 5.0 Hz, H-B), 3.26 (dd, 1H, $J_{B,C}$ = 5.0; $J_{C,D}$ = 17.2 Hz, H-C), 2.75 (d, 1H, $J_{C,D}$ = 17.2 Hz, H-D), 2.48–2.52 (m, 1H, H-E), 2.32 (s, 3H, -CH_3), 2.15 (d, 1H, $J_{E,F}$ = 10.4 Hz, H-F); ^{13}C NMR ($CDCl_3$; 150 MHz) δ/ppm: 150.62 (s), 144.54 (s), 142.08 (s), 139.71 (s), 136.61 (s), 133.97 (s), 130.57 (s), 128.83 (d, d), 126.30 (d), 125.99 (d), 125.85 (d), 125.53 (d, d), 123.35 (d), 123.10 (d), 120.64 (d), 120.35 (d), 41.83 (d, C_A), 41.63 (t, $C_{E,F}$), 40.03 (d, C_B), 31.37 (t, $C_{C,D}$), 20.70 (q); IR ν_{max}/cm^{-1} 2940, 1668, 1458, 1264, 950, 821; HRMS: M^+_{calcd} 328.1286; M^+_{found} 328.1292.

4-[(*Z*)-2-(4-Methoxyphenyl)ethenyl]-5-thiatetracyclo[6.6.1.0^{2,6}.0^{9,14}]pentadeca-2(6),3,9(14),10,12-pentaene (*cis*-4): Yield 16 %, R_f 0.55 (petroleum ether / diethyl ether 10:1); colourless crystals; mp 138 – 141 °C; UV (96% EtOH) λ_{max}/nm (ε/dm^3 mol^{-1} cm^{-1}): 322 (10472), 246 (12724, sh); ^{1}H NMR ($CDCl_3$, 600 MHz) δ/ppm: 7,24 (d, 2H, J = 8,5 Hz, H-ar), 7,01-7,08 (m, 4H, H-ar), 6,84 (d, 2H, J = 8,5 Hz, H-ar), 6,72 (s, 1H, H-t), 6,49 (d, 1H, J = 11,7 Hz, H-et), 6,36 (d, 1H, J = 11,7 Hz, H-et), 3,84 (d, 1H, $J_{A,E}$ = 4,8 Hz, H-A), 3,82 (s, 3H, H-OCH_3), 3,51 (t, 1H, $J_{B,C}$ = 5,6 Hz, H-B), 3,13 (dd, 1H, $J_{B,C}$ = 5,6; $J_{C,D}$ = 16,8 Hz, H-C), 2,62 (d, 1H, $J_{C,D}$ = 16,8 Hz, H-D), 2,43–2,48 (m, 1H, H-E), 2,08 (d, 1H, $J_{E,F}$ = 10,4 Hz, H-F); ^{13}C NMR ($CDCl_3$, 150 MHz) δ/ppm: 150.69 (s), 150.64 (s), 144 (s), 144 (s), 140.79 (s), 139 (s), 129.56 (d), 129.33 (s), 126.80 (d), 126.40 (d), 125.89 (d), 125.73 (d), 123.03 (d), 122.52 (d), 120.33 (d), 120.29 (d), 113.63 (d), 113.31 (d), 54.70 (q), 41.58 (t), 40.04 (d), 39.97 (d), 31.12 (t); IR ν_{max}/cm^{-1} 2933, 1601, 1509, 1247, 824.

4-[(*E*)-2-(4-Methoxyphenyl)ethenyl]-5-thiatetracyclo[6.6.1.0^{2,6}.0^{9,14}]pentadeca-2(6),3,9(14),10,12-pentaene (*trans*-4): Yield 61 %, R_f 0.41 (petroleum ether / diethyl ether 10:1); colourless crystals; mp 143 – 145 °C; UV (96% EtOH) λ_{max}/nm (ε/dm^3 mol^{-1} cm^{-1}): 347 (22030), 260 (10759, sh), 208 (26880); ^{1}H NMR ($CDCl_3$, 600 MHz) δ/ppm: 7.33 (d, 2H, J = 9.0 Hz, H-ar), 7.30 (d, 1H, J = 7.3 Hz, H-ar), 7.11 (d, 1H, J = 7.3 Hz, H-ar), 7.08 (dt, 1H, J = 1.4; 7.3 Hz, H-ar), 7.04 (dt, 1H, J = 1.4; 7.3 Hz, H-ar), 6.95 (d, 1H, J = 16.3 Hz, H-et), 6.84 (d, 2H, J = 9.0 Hz, H-ar), 6.75 (s, 1H, H-t), 6.69 (d, 1H, J = 16.3 Hz, H-et), 3.90 (d, 1H, $J_{A,E}$ = 4.4 Hz, H-A), 3.80 (s, 3H, -OCH_3), 3.56 (t, 1H, $J_{B,C}$ = 6.2 Hz, H-B), 3.25 (dd, 1H, $J_{B,C}$ = 6.2; $J_{C,D}$ = 16.9 Hz, H-C), 2.74 (d, 1H, $J_{C,D}$ = 16.9 Hz, H-D), 2.47–2.52 (m, 1H, H-E), 2.15 (d, 1H, $J_{E,F}$ = 9.7 Hz, H-F); ^{13}C NMR ($CDCl_3$; 150 MHz) δ/ppm: 158.59 (s), 150.64 (s), 144.55 (s), 142.03 (s), 139.84 (s), 130.21 (s), 129.59 (s), 126.80 (d, d), 125.96 (d), 125.95 (d), 125.82 (d), 123.08 (d), 122.99 (d), 120.33 (d), 119.63 (d), 113.64 (d, d), 54.80 (q), 41.84 (d, C_A), 41.63 (t, $C_{E,F}$), 40.04 (d, C_B), 31.35 (t, $C_{C,D}$); IR ν_{max}/cm^{-1} 2933, 1601, 1509, 1247, 824 cm^{-1}; HRMS: M^+_{calcd} 344.1235; M^+_{found} 344.1266.

4-[(*E*)-2-(2-Methylphenyl)ethenyl]-5-thiatetracyclo[6.6.1.0^{2,6}.0^{9,14}]pentadeca-2(6),3,9(14),10,12-pentaene (*trans*-5): Yield 95 %, R_f 0.77 (petroleum ether / diethyl ether 10:1); colourless oil; UV (96% EtOH) λ_{max}/nm (ε/dm^3 mol^{-1} cm^{-1}): 340 (13304), 275 (5139, sh), 235 (10968); ^{1}H NMR ($CDCl_3$, 300 MHz) δ/ppm: 7.48 (d, 1H, J = 7.0 Hz, H-ar), 7.31 (d, 1H, J = 7.0 Hz, H-ar), 7.10 – 7.20 (m, 4H, H-ar), 7.07 (dt, 2H, J = 7.0; 1.4 Hz, H-ar), 7.00 (d, 1H, J = 16.2 Hz, H-et), 6.93 (d, 1H, J = 16.2 Hz, H-et), 6.81 (s, 1H, H-t), 3.92 (d, 1H, J = 4.5 Hz, H-A), 3.58 (t, 1H, J = 5.0 Hz, H-B), 3.27 (dd, 1H, J = 17.2; 5.0 Hz, H-C), 2.76 (d, 1H, J = 17.2 Hz, H-D), 2.46 – 2.56

(m, 1H, H-E), 2.16 (d, 1H, J = 10.5 Hz, H-F); ^{13}C NMR ($CDCl_3$, 75 MHz) δ/ppm: 151.11 (s), 145.01 (s), 142.66 (s), 140.32 (s), 136.19 (s), 135.51 (s), 131.38 (s), 130.38 (d), 127.23 (d), 126.52 (d), 126.39 (d), 126.15 (d), 124.95 (d), 124.60 (d), 124.19 (d), 123.63 (d), 123.22 (d), 120.88 (d), 42.32 (d), 42.13 (t), 40.51 (d), 31.89 (t), 19.87 (q); IR ν_{max}/cm^{-1} 2219, 1472, 937; HRMS: M^+_{calcd} 328.1286; M^+_{found} 328.1288.

2-[(*E*)-2-{5-Thiatetracyclo[6.6.1.0^{2,6}.0^{9,14}]pentadeca-2(6),3,9(14),10,12-pentaen-4-yl}ethenyl]benzonitrile (*trans*-6): Yield 72 %, R_f 0.64 (petroleum ether / diethyl ether 10:1); colourless crystals; mp 159 – 161 °C; UV (96% EtOH) λ_{max}/nm (ε/dm^3 mol^{-1} cm^{-1}): 357 (16592), 263 (10048, sh); ^{1}H NMR ($CDCl_3$, 300 MHz) δ/ppm: 7.65 (d, 1H, J = 7.9 Hz, H-ar), 7.59 (dd, 1H, J = 7.9; 1.7 Hz, H-ar), 7.51 (dt, 2H, J = 7.9; 1.3 Hz, H-ar), 7.20 – 7.34 (m, 4H, H-ar/ H-et), 7.04-7.16 (m, 3H, H-ar/H-et), 6.94 (s, 1H, H-t), 3.94 (d, 1H, J = 4.5 Hz, H-A), 3.59 (t, 1H, J = 5.1 Hz, H-B), 3.29 (dd, 1H, J = 17.2; 5.1 Hz, H-C), 2.78 (d, 1H, J = 17.2 Hz, H-D), 2.46 – 2.58 (m, 1H, H-E), 2.15 (d, 1H, J = 10.4 Hz, H-F); ^{13}C NMR ($CDCl_3$, 150 MHz) δ/ppm: 155.01 (s), 150.42 (s), 148.28 (s), 144.39 (s), 144.37 (s), 144.31 (s), 140.04 (s), 132.67 (d), 132.09 (d), 126.47 (d), 126.06 (d), 125.98 (d), 125.93 (d), 125.22 (d), 124.38 (d), 123.16 (d), 121.21 (d), 120.41 (d), 41.79 (d), 41.55 (t), 39.90 (d), 31.48 (t); IR ν_{max}/cm^{-1} 2219, 1472, 937; HRMS: M^+_{calcd} 339.1082; M^+_{found} 339.1099.

4-[(*E*)-2-(4-Methylphenyl)ethenyl]-3-thiatetracyclo[6.6.1.0^{2,6}.0^{9,14}]pentadeca-2(6),4,9,11,13-pentaene (*trans*-7): Yield 46 %, R_f 0.75 (petroleum ether / diethyl ether 10:1); colourless oil; UV (96% EtOH) λ_{max}/nm (ε/dm^3 mol^{-1} cm^{-1}): 341 (23135), 234 (14183, sh); ^{1}H NMR ($CDCl_3$, 300 MHz) δ/ppm: : 7.06 – 7.35 (m, 8H, H-ar), 7.02 (d, 1H, J = 16.0 Hz, H-et), 6.74 (d, 1H, J = 16.0 Hz, H-et), 6.59 (s, 1H, H-t), 3.98 (d, 1H, J = 4.6 Hz, H-A), 3.55 (t, 1H, J = 5.0 Hz, H-B), 3.11 (dd, 1H, J = 17.0; 5.0 Hz, H-C), 2.60 (d, 1H, J = 17.0 Hz, H-D), 2.45 – 2.56 (m, 1H, H-E), 2.23 (d, 1H, J = 10.3 Hz, H-F); ^{13}C NMR ($CDCl_3$, 150 MHz) δ/ppm: 149.66 (s), 144.84 (s), 139.19 (s), 138.48 (s), 136.58 (s), 133.99 (s), 130.43 (s), 128.83 (2d), 126.93 (d), 126.12 (d), 126.08 (d), 126.02 (d), 125.52 (2d), 123.11 (d), 120.71 (d), 120.41 (d), 41.87 (t), 41.27 (d), 39.39 (d), 31.54 (t), 20.69 (q); IR ν_{max}/cm^{-1} 1685, 1540; HRMS: M^+_{calcd} 328.1286; M^+_{found} 328.1295.

Synthesis of compounds 8–11

The cyclization reactions were performed by irradiation of a toluene solution (3.7×10^{-3} mol dm^{-3}) of the mixture of isomers of compounds **3**–**7** in the presence of iodine (1 mg) at 350 nm in a Rayonet

reactor (16 lamps) in a quartz vessel for 18 h, respectively. After chromatographic purification on silica gel using petroleum ether as the eluent, the photoproducts **8**–**11** (from **3**–**5** and **7**) were isolated. In the case of the cyano-substituted derivative **6**, the expected cyclization photoproduct was not formed, whereby the mixture of the starting *cis*- and *trans*-isomers were the only isolated products.

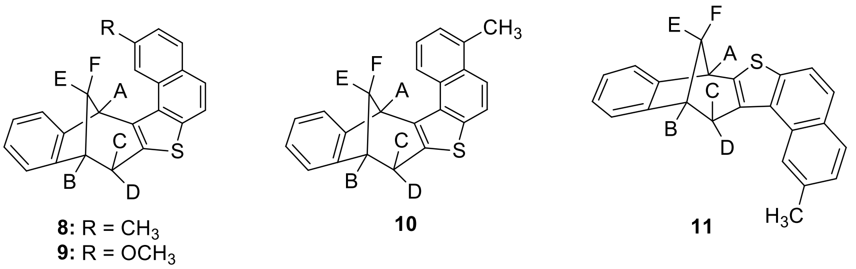

6-Methyl-13-thiahexacyclo[14.6.1.0^{2,14}.0^{3,12}.0^{4,9}.0^{17,22}]tricosa-2(14),3(12),4(9),5,7,10,17(22), 18,20-nonaene (8): Yield 23 %, R_f 0.76 (petroleum ether / diethyl ether 10:1); colourless crystals; mp 146 – 150 °C; UV (96% EtOH) λ_{max}/nm (ε/dm^3 mol^{-1} cm^{-1}): 340 (3591), 313 (8297), 266 (17533, sh), 231 (50403), 207 (96514); ^{1}H NMR ($CDCl_3$, 600 MHz) δ/ppm: 8.89 (s, 1H, H-ar), 7.83 (d, 1H, J = 8.2 Hz, H-ar), 7.65 (d, 1H, J = 8.6 Hz, H-ar), 7.56 (d, 1H, J = 8.6 Hz, H-ar), 7.36 – 7.40 (m, 3H, H-ar), 7.11 (t, 1H, J = 7.4 Hz, H-ar), 7.03 (t, 1H, J = 7.4 Hz, H-ar), 5.24 (d, 1H, $J_{A,E}$ = 4.2 Hz, H-A), 3.65 (t, 1H, $J_{B,C}$ = 5.3 Hz, H-B), 3.53 (dd, 1H, $J_{B,C}$ = 5.3; $J_{C,D}$ = 16.8 Hz, H-C), 2.97 (d, 1H, $J_{C,D}$ = 16.8 Hz, H-D), 2.71 (s, 3H, -CH_3), 2.67 – 2.70 (m, 1H, H-E), 2.30 (d, 1H, $J_{E,F}$ = 11.3 Hz, H-F); ^{13}C NMR ($CDCl_3$, 75 MHz) δ/ppm: 128.98 (d), 126.57 (d, d), 126.52 (d), 123.94 (d), 123.89 (d), 122.81 (d), 120.84 (d), 120.01 (d), 42.56 (d, C_A), 42.52 (t, $C_{E,F}$), 39.70 (d, C_B), 33.01 (t, $C_{C,D}$); IR ν_{max}/cm^{-1} 2924, 1623, 1468, 1138, 827; HRMS: M^+_{calcd} 326.1129; M^+_{found} 326.1130.

6-Methoxy-13-thiahexacyclo[14.6.1.0^{2,14}.0^{3,12}.0^{4,9}.0^{17,22}]tricosa-2(14),3(12),4(9),5,7,10,17(22), 18,20-nonaene (9): Yield 37 %, R_f 0.52 (petroleum ether / diethyl ether 10:1); colourless crystals; mp 168 – 171 °C; UV (96% EtOH) λ_{max}/nm (ε/dm^3 mol^{-1} cm^{-1}): 345 (6033), 330 (4979), 309 (10496), 297 (8257, sh), 267 (13261), 251 (41386), 237 (32840, sh); ^{1}H NMR ($CDCl_3$, 600 MHz) δ/ppm: 8.48 (s, 1H, H-ar), 7.85 (d, 1H, J = 7.7 Hz, H-ar), 7.60 (d, 1H, J = 8.7 Hz, H-ar), 7.55 (d, 1H, J = 8.7 Hz, H-ar), 7.39 (d, 2H, J = 7.7 Hz, H-ar), 7.20 – 7.23 (m, 1H, H-ar), 7,12 (t, 1H, J = 7.1 Hz, H-ar), 7.05 (t, 1H, J = 7.1 Hz, H-ar), 5.18 (d, 1H, $J_{A,E}$ = 5.0 Hz, H-A), 4.12 (s, 3H, -OCH_3), 3.66 (t, 1H, $J_{B,C}$ = 5.8 Hz, H-B), 3.54 (dd, 1H, $J_{B,C}$ = 5.8; $J_{C,D}$ = 17.1 Hz, H-C), 2.98 (d, 1H, $J_{C,D}$ = 17.1 Hz, H-D), 2.67–2,75 (m, 1H, H-E), 2.32 (d, 1H, $J_{E,F}$ = 10.0 Hz, H-F); ^{13}C NMR ($CDCl_3$, 75

MHz) δ/ppm: 157.69 (s), 150.54 (s), 148.04 (s), 145.48 (s), 144.74 (s), 138.71 (s), 138.06 (s), 133.10 (s), 131.11 (s), 130.53 (d), 126.59 (2d), 123.96 (d), 123.92 (d), 120.94 (d), 118.68 (d), 115.58 (d), 104.14 (d), 55.59 (q), 42.69 (d), 42.54 (t), 39.69 (d), 33.00 (t); IR ν_{max}/cm^{-1} 2932, 1621, 1514, 754; HRMS: M^+_{calcd} 342.1078; M^+_{found} 342.1080.

8-Methyl-13-thiahexacyclo[14.6.1.0^{2,14}.0^{3,12}.0^{4,9}.0^{17,22}]tricosa-2(14),3(12),4(9),5,7,10,17 (22),18,20-nonaene) (10): Yield 42 %, R_f 0.76 (petroleum ether / diethyl ether 10:1); colourless oil; UV (96% EtOH) λ_{max}/nm (ε/dm^3 mol^{-1} cm^{-1}): 325 (14944), 263 (14130, sh), 236 (25089); ^{1}H NMR ($CDCl_3$, 300 MHz) δ/ppm: 9.01 (d, 1H, J = 9.0 Hz, H-ar), 7.00 – 7.80 (m, 8H, H-ar), 5.23 (d, 1H, $J_{A,E}$ = 4.9 Hz, H-A), 3.63 (t, 1H, $J_{B,C}$ = 5.1 Hz, H-B), 3.53 (dd, 1H, $J_{B,C}$ = 5.1; $J_{C,D}$ = 16.7 Hz, H-C), 2.97 (d, 1H, $J_{C,D}$ = 16.7 Hz, H-D), 2.75 (s, 3H, -CH_3), 2.64 – 2.72 (m, 1H, H-E), 2.28 (d, 1H, $J_{E,F}$ = 9.9 Hz, H-F); ^{13}C NMR ($CDCl_3$, 75 MHz) δ/ppm: 151.15 (s), 151.10 (s), 145.47 (s), 145.01 (s), 142.66 (s), 140.32 (s), 136.18 (s), 135.51 (s), 131.38 (s) 130.38 (d), 127.22 (d), 126.54 (d), 126.52 (d), 126.38 (d), 124.60 (d), 123.63 (d), 123.22 (d), 120.87 (d) 42.31 (d), 42.13 (t), 40.50 (d), 31.89 (t), 20.65 (q); IR ν_{max}/cm^{-1} 2932, 1514, 1137, 828; HRMS: M^+_{calcd} 326.1129; M^+_{found} 326.1129.

10-Methyl-3-thiahexacyclo[14.6.1.0^{2,14}.0^{3,12}.0^{4,9}.0^{17,22}]tricosa-2(14),4(13),5,7(12),8,10,17(22), 18,20-nonaene (11): due to its instability, it was not possible to characterize the compound completely; Yield 28 %, R_f 0.76 (petroleum ether / diethyl ether 10:1); colourless oil; ^{1}H NMR ($CDCl_3$, 300 MHz) δ/ppm: 7.77 (d, 1H, J = 7.8 Hz, H-ar), 7.67 (d, 1H, J = 7.8 Hz, H-ar), 7.55 (d, 1H, J = 7.8 Hz, H-ar), 7.30 – 7.40 (m, 3H, H-ar), 7.24 (s, 1H, H-ar), 7.16 – 7.22 (m, 1H, H-ar), 7.08 (dd, 1H, J = 6.8; 1.8 Hz, H-ar), 4.15 (d, 1H, $J_{A,E}$ = 4.1 Hz, H-A), 3.76 – 3.87 (m, 2H, H-B, H-C), 3.34 (d, 1H, $J_{C,D}$ = 14.8 Hz, H-D), 2.65 – 2.74 (m, 1H, H-E), 2.40 (d, 1H, $J_{E,F}$ = 10.3 Hz, H-F), 1.80 (s, 3H, -CH_3); ^{13}C NMR ($CDCl_3$, 75 MHz) δ/ppm: 156.49 (s), 150.26 (s), 150.09 (s), 149.87 (s), 146.20 (s), 145.92 (s), 142.04 (s), 137.26 (s), 135.27 (s), 128.56 (d), 126.74 (d), 126.57 (d), 126.53 (d), 123.82 (d), 123.51 (d), 123.16 (d), 120.99 (d), 119.91 (d), 42.46 (d), 42.71 (t), 40.12 (d), 38.15 (t), 31.24 (q); IR ν_{max}/cm^{-1} 2945, 1586, 1137, 970; HRMS: M^+_{calcd} 326.1129; M^+_{found} 326.1124.

Synthesis of compound 12

To a stirred solution of aldehyde **1** (0.417 mmol) in absolute ethanol (0.4 mL), an aqueous solution of sodium hydroxide (0.025 g, 0.625 mmol in 1 mL of distilled water) and acetone (0.1 mL) was

added dropwise. After one hour of stirring at rt, the reaction mixture was neutralized with 20% acetic acid (0.2 mL) and worked up with water and toluene (4 × 5 mL). The toluene extracts were dried (anhydrous $MgSO_4$), concentrated and the crude mixture purified by column chromatography on silica gel using petroleum ether as the eluent, to afford product **12**.

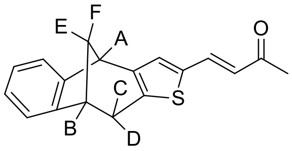

12

(3*E*)-4-{5-Thiatetracyclo[6.6.1.0^{2,6}.0^{9,14}]pentadeca-2(6),3,9(14),10,12-pentaen-4-yl}but-3-en-2-one (12): Yield 23%, R_f 0.22 (petroleum ether / diethyl ether 10:1); colourless oil; UV (96% EtOH) λ_{max}/nm (ε/dm^3 mol^{-1} cm^{-1}): 357 (17847), 286 (6308), 228 (10755); ^{1}H NMR ($CDCl_3$, 300 MHz) δ/ppm: 7.49 (d, 1H, J = 15.6 Hz, H-et), 7.04-7.32 (m, 4H, H-ar), 7.02 (s, 1H, H-t), 6.34 (d, 1H, J = 15.6 Hz, H-et), 3.95 (d, 1H, $J_{A,E}$ = 4.6 Hz, H-A), 3.59 (t, 1H, $J_{B,C}$ = 4.6 Hz, H-B), 3.28 (dd, 1H, $J_{B,C}$ = 4,6; $J_{C,D}$ = 17.2 Hz, H-C), 2.78 (d, 1H, $J_{C,D}$ = 17.2 Hz, H-D), 2.45–2.59 (m, 1H, H-E), 2.28 (s, 3 H, H-CH_3), 2.13 (d, 1H, $J_{E,F}$ = 11.1 Hz, H-F); ^{13}C NMR ($CDCl_3$, 75 MHz) δ/ppm: 197.71 (s), 150.68 (s), 144.72 (s), 143.72 (s), 137.53 (s), 136.14 (d), 129.69 (d), 126.69 (d), 126.64 (d), 124.18 (d), 123.73 (d), 121.01 (d), 42.18 (d), 41.94 (t), 40.22 (d), 32.17 (t), 27.58 (q); IR ν_{max}/cm^{-1} 2945, 1662, 1254, 753; HRMS: M^+_{calcd} 280.0922; M^+_{found} 280.0949.

X-ray Crystallography

Single crystal measurements were performed on an Enraf Nonius CAD4 diffractometer, using graphite monochromated MoKα (0.71073 Å) radiation at room temperature [293(2) K]. The WinGX standard procedure was applied for data reduction [4]. Three standard reflections were measured every 120 minutes as intensity control. No absorption correction was applied.

The structure was solved with SHELXS97 [5] and refined with SHELXL2018 [6]. The model was refined using the full matrix least squares refinement; all non-hydrogen atoms were refined anisotropically. Hydrogen atoms were located in a difference Fourier map and refined as a mixture of free and riding entities. Molecular geometry calculations were performed by PLATON [7], and the molecular graphics were prepared using ORTEP-3 [8] and CCDC-Mercury [9]. Crystallographic and structure refinement data for the structure reported in this paper are shown in Table 1.

Supplementary crystallographic data for this paper can be obtained free of charge via www.ccdc.cam.ac.uk/conts/retrieving.html (or from the Cambridge Crystallographic Data Centre, 12, Union Road, Cambridge CB2 1EZ, UK; fax: +44 1223 336033; or deposit@ccdc.cam.ac.uk). CCDC-1981794 contains the supplementary crystallographic data for this paper.

[4] Harms, K.; Wocadlo, S. XCAD-4, Program for Processing CAD4 Diffractometer Data, University of Marburg, Germany, 1995.
[5] Farrugia, L. J. J. Appl. Cryst. 1999, 32, 837–838.
[6] Sheldrick, G. M. Acta Crystallogr. A, 2008, 64, 112-122.
[7] Spek, A. L. Acta Crystallogr. D, 2009, D65, 148-155.
[8] Farrugia, L. J. ORTEP-3 for Windows, J. Appl. Cryst. 1997, 30, 565–565.
[9] Macrae, C. F.; Edgington, P. R.; McCabe, P.; Pidcock, E.; Shields, G.; Taylor, R.; Towler M.; van de Streek, J. J. Appl. Cryst. 2006, 39, 453-457.

Table S1: Crystallographic data and structure refinement details for compound *trans*-**6.**

Compound	*trans*-**6**
Empirical formula	$C_{23}H_{17}NS$
Formula wt. / g mol^{-1}	339.44
Crystal dimensions / mm	0.14 x 0.10 x 0.08
Space group	$P\bar{1}$
a / Å	8.0604(12)
b / Å	8.8620(13)
c / Å	13.723(2)
α / °	87.156(13)
β / °	73.759(13)
γ / °	75.094(13)
Z	2
V / Å^3	909.2(2)
D_{calc} / g cm^{-3}	1.240
μ / mm^{-1}	0.182
Θ range / °	8.48 – 15.73
T(K)	293(2)
Radiation wavelength	0.71073 (MoK_α)
Diffractometer type	Enraf Nonius CAD4
Range of h, k, l	$-9 > h > 0$; $-10 > k > 10$; $-16 > l > 15$
Reflections collected	3765
Independent reflections	3191
Observed reflections ($I \geq 2\sigma$)	2067
R_{int}	0.0926
R (F)	0.0737
R_w (F^2)	0.2058
No. of parameters, restraints	266, 0
Goodness of fit	1.052
$\Delta\rho_{max}$, $\Delta\rho_{min}$ (eÅ^{-3})	0.616; –0.326

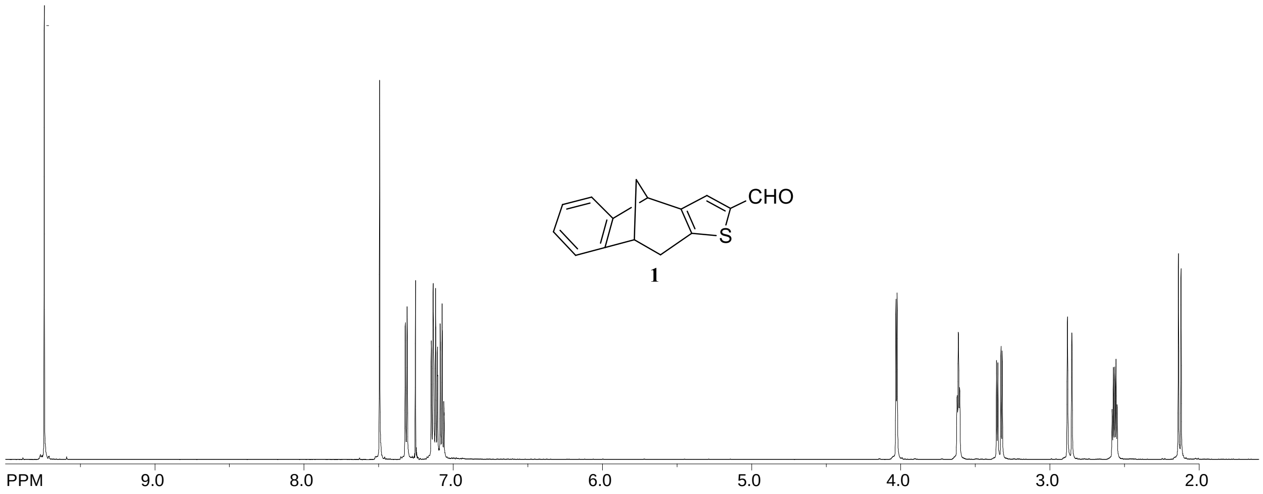

Figure S1: ^{1}H NMR spectrum ($CDCl_3$, 600 MHz) of compound **1**

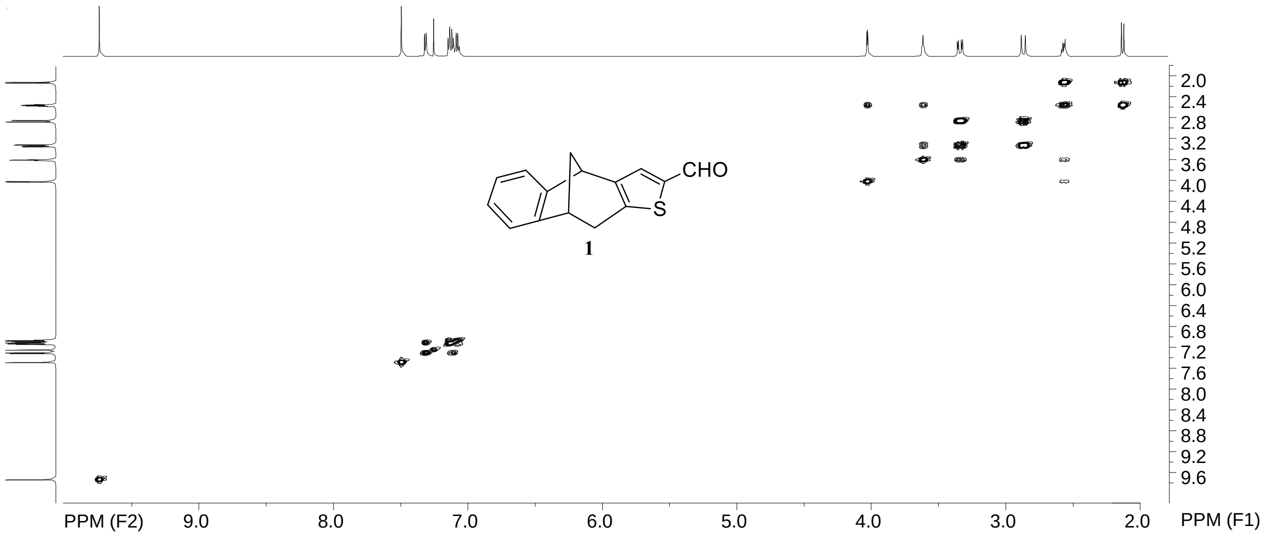

Figure S2: COSY spectrum ($CDCl_3$, 600 MHz) of compound **1**

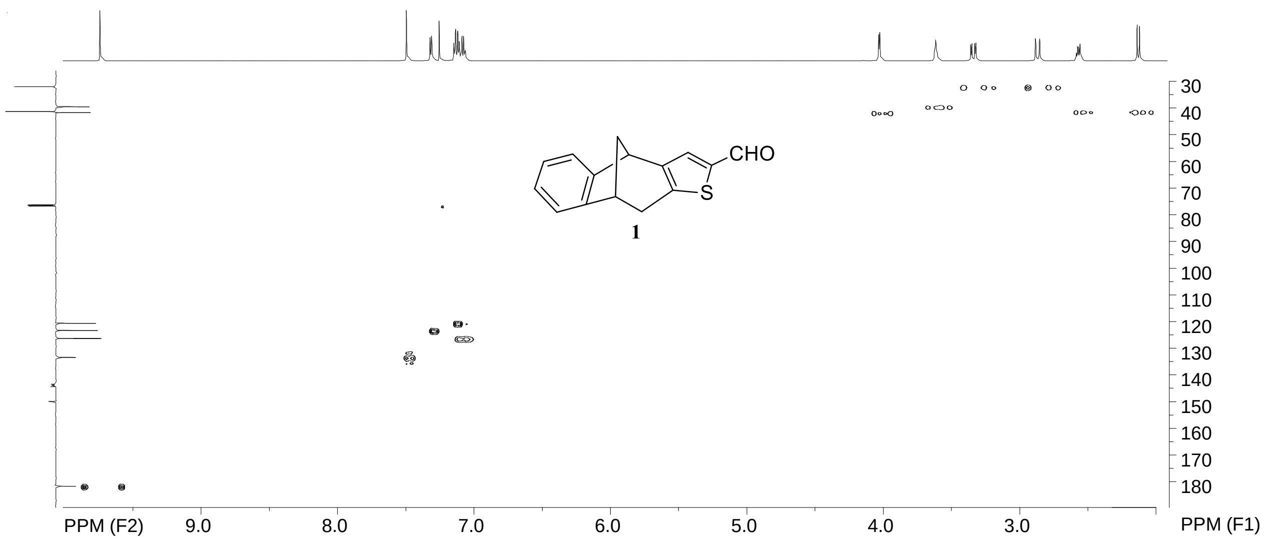

Figure S3: HETCOR spectrum ($CDCl_3$, 600 MHz) of compound **1**

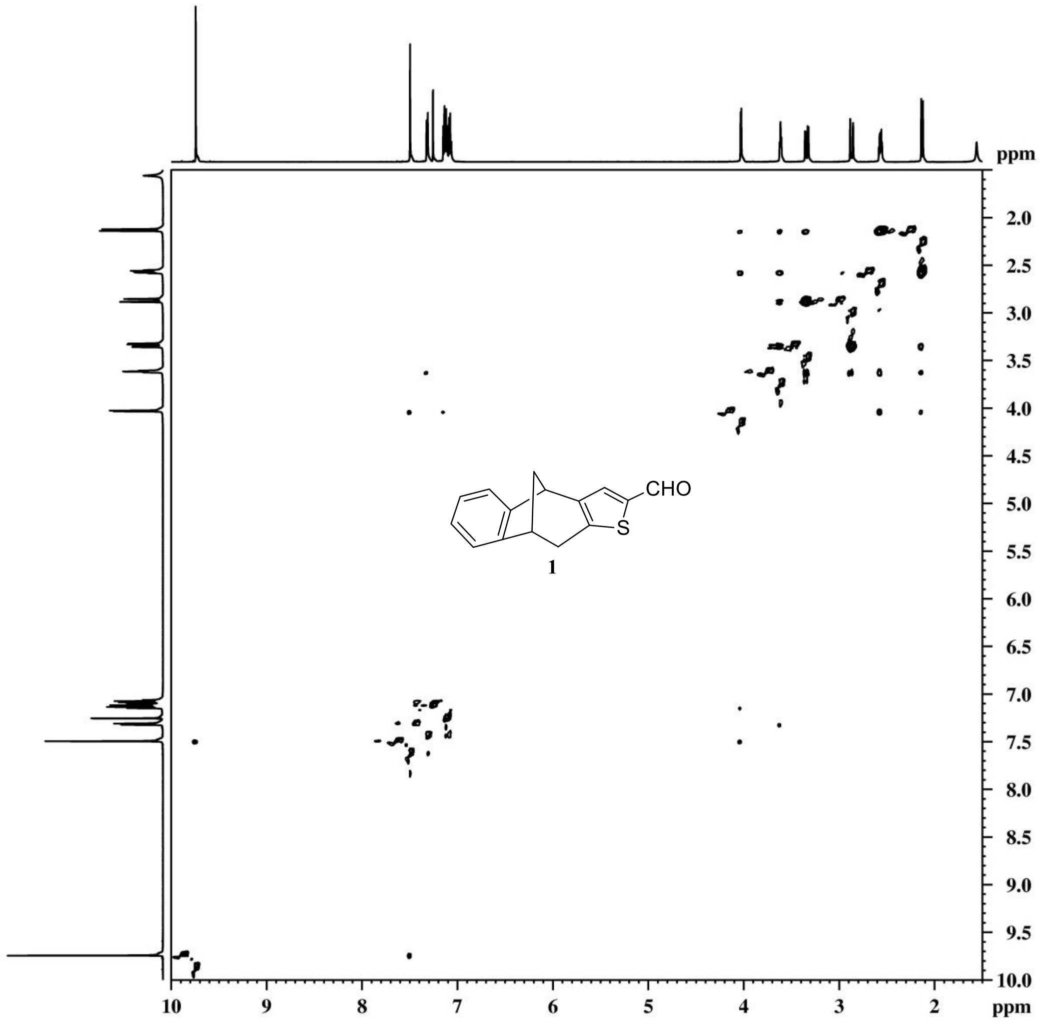

Figure S4: NOESY spectrum ($CDCl_3$, 600 MHz) of compound **1**

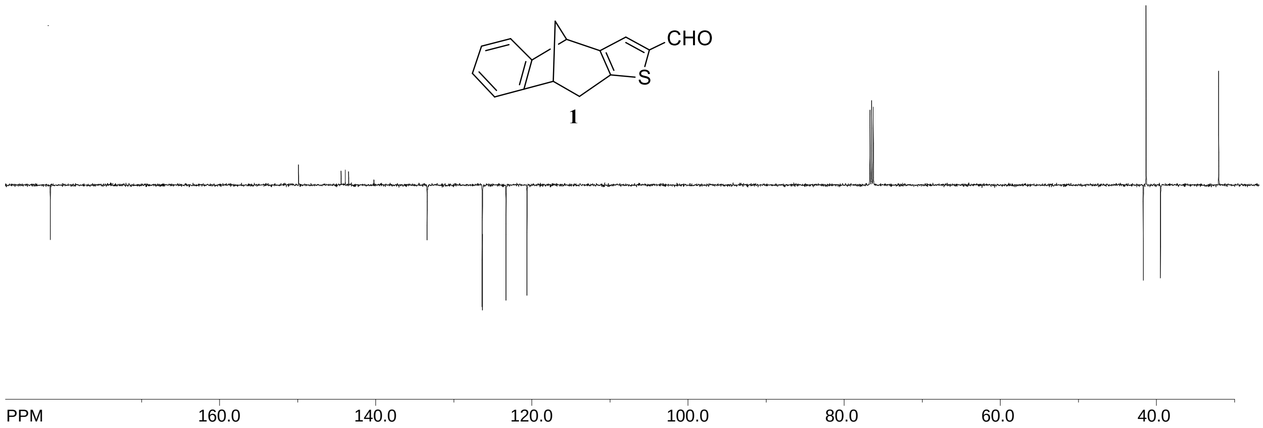

Figure S5: ^{13}C NMR spectrum ($CDCl_3$, 150 MHz) of compound **1**

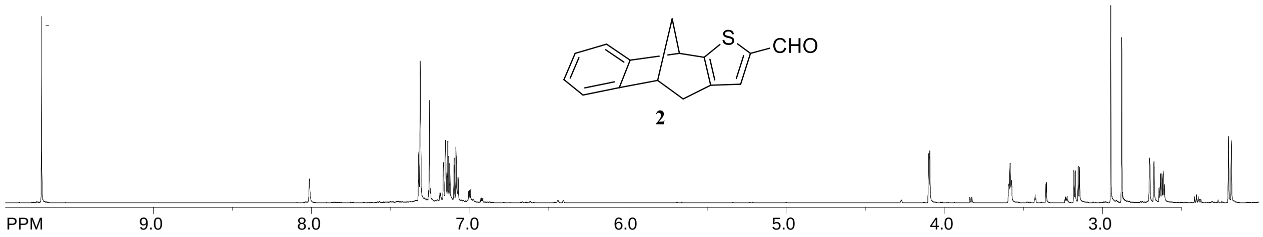

Figure S6: ^{1}H NMR spectrum ($CDCl_3$, 600 MHz) of compound **2**

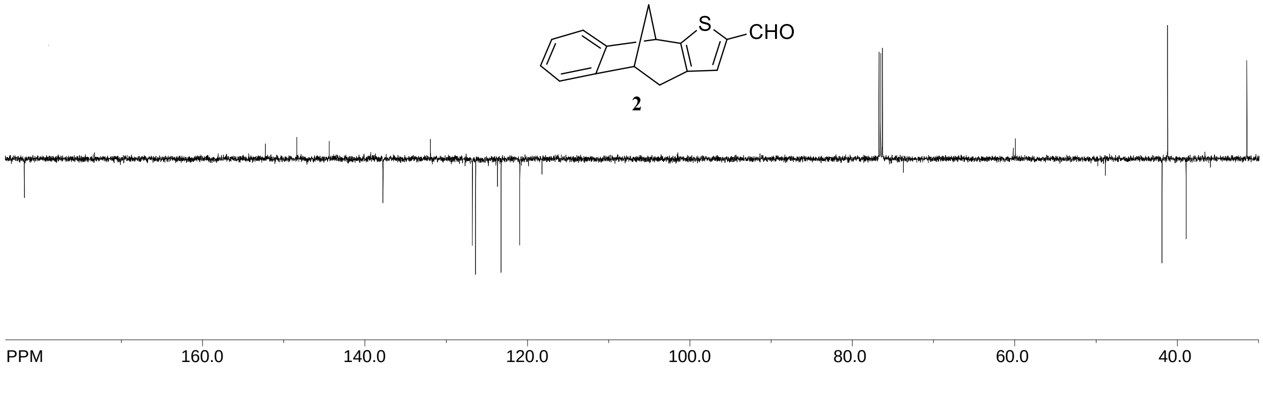

Figure S7: ^{13}C NMR spectrum ($CDCl_3$, 150 MHz) of compound **2**

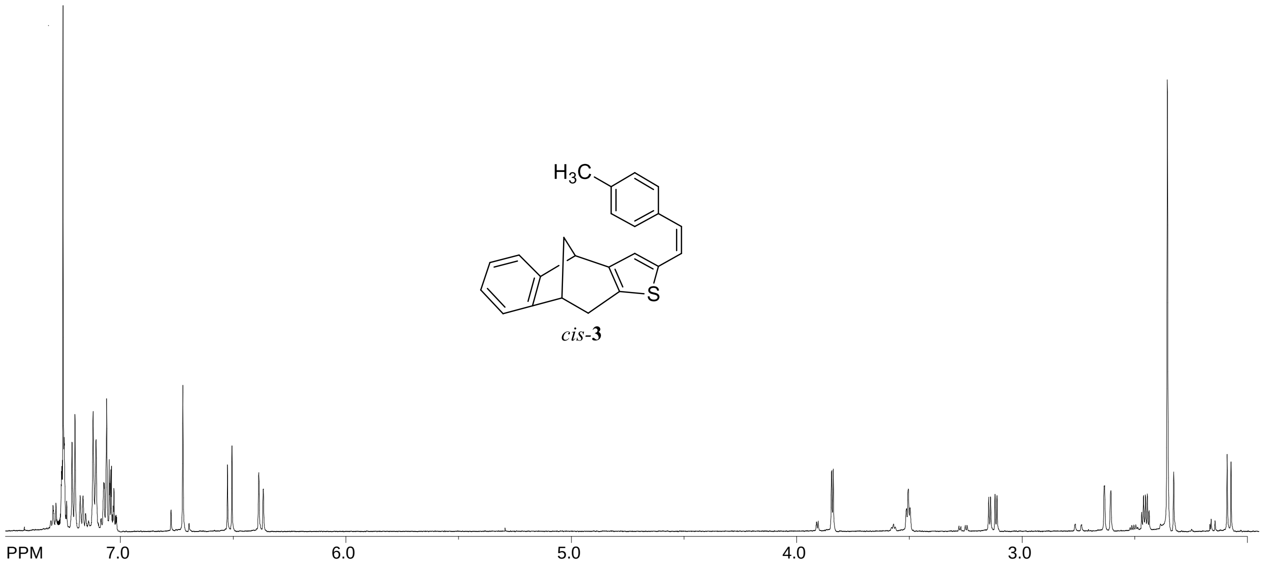

Figure S8: ^{1}H NMR spectrum ($CDCl_3$, 600 MHz) of compound *cis*-**3**

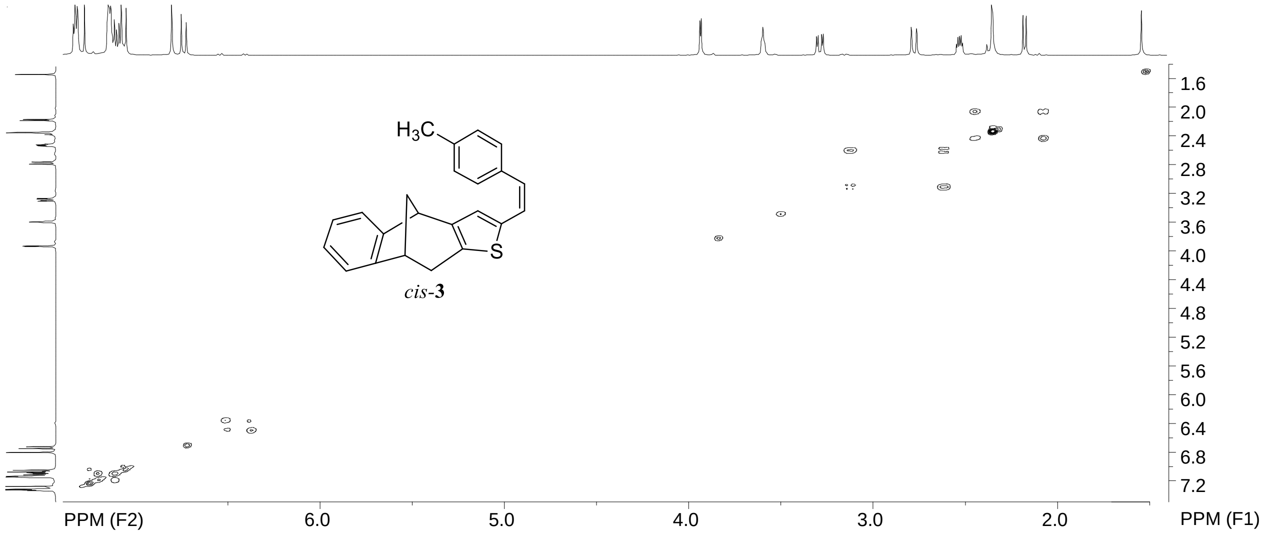

Figure S9: COSY spectrum ($CDCl_3$, 600 MHz) of compound *cis*-**3**

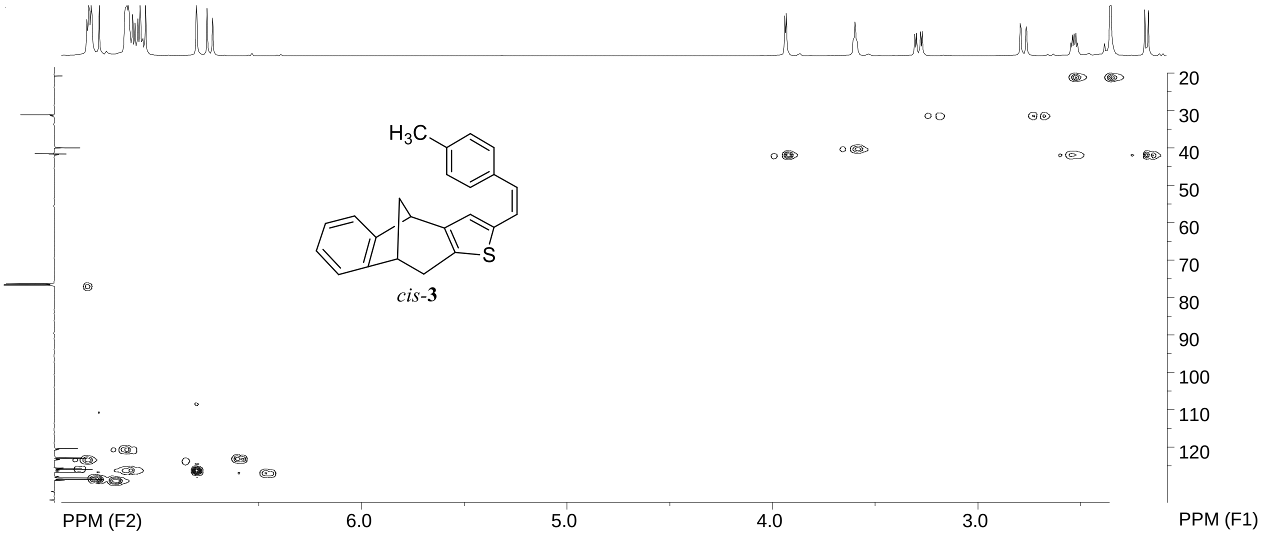

Figure S10: HETCOR spectrum ($CDCl_3$, 600 MHz) of compound *cis*-**3**

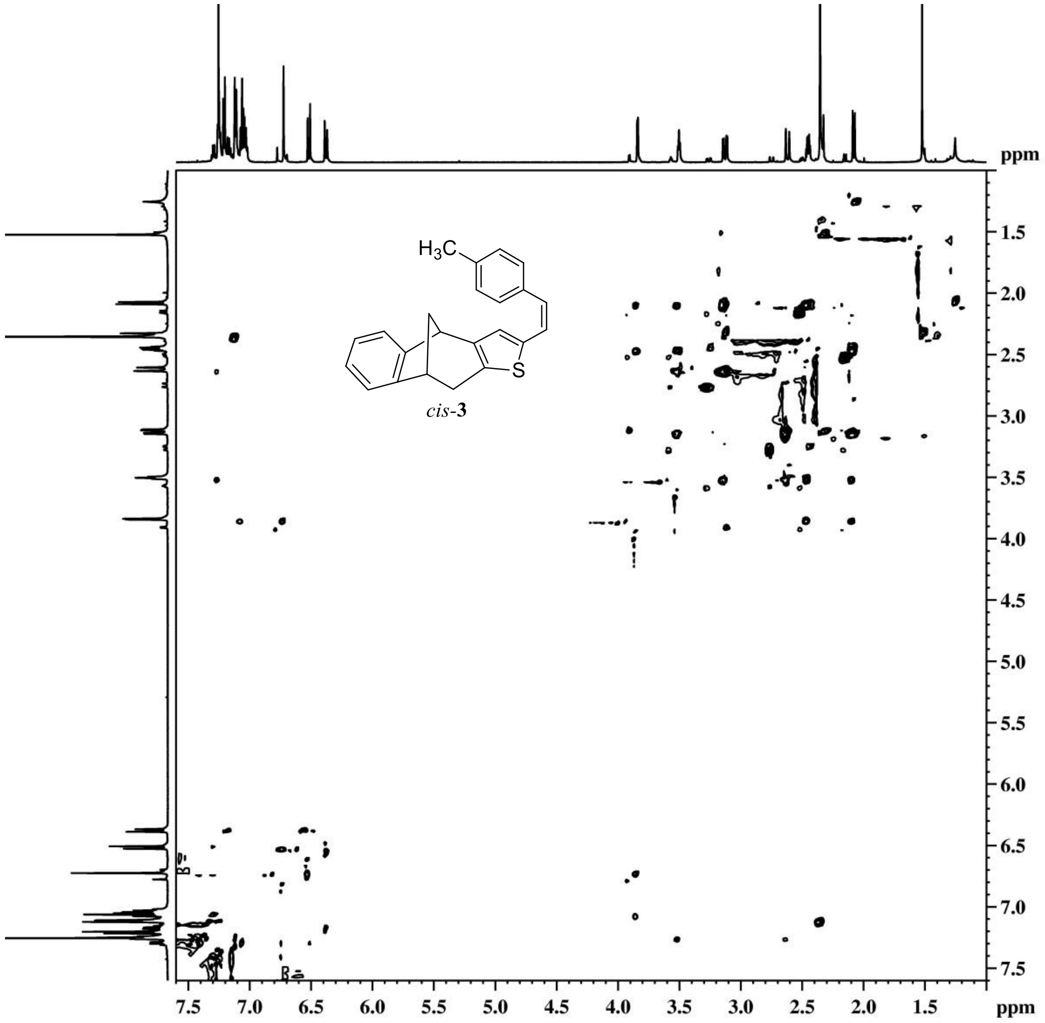

Figure S11: NOESY spectrum ($CDCl_3$, 600 MHz) of compound *cis*-**3**

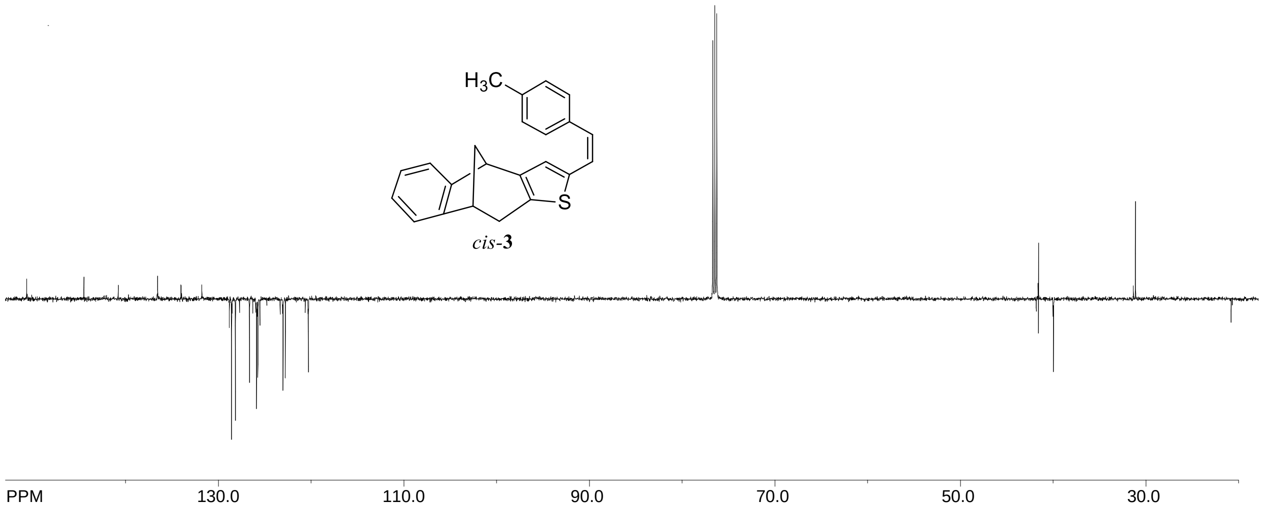

Figure S12: ^{13}C NMR spectrum ($CDCl_3$, 150 MHz) of compound *cis*-**3**

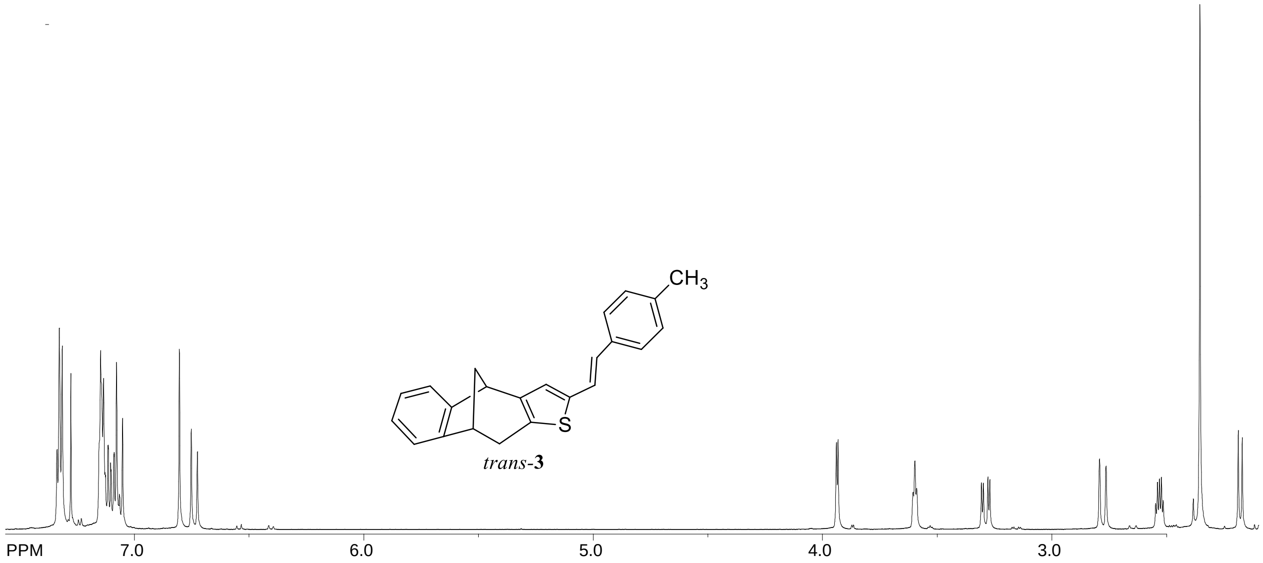

Figure S13: ^{1}H NMR spectrum ($CDCl_3$, 600 MHz) of compound *trans*-**3**

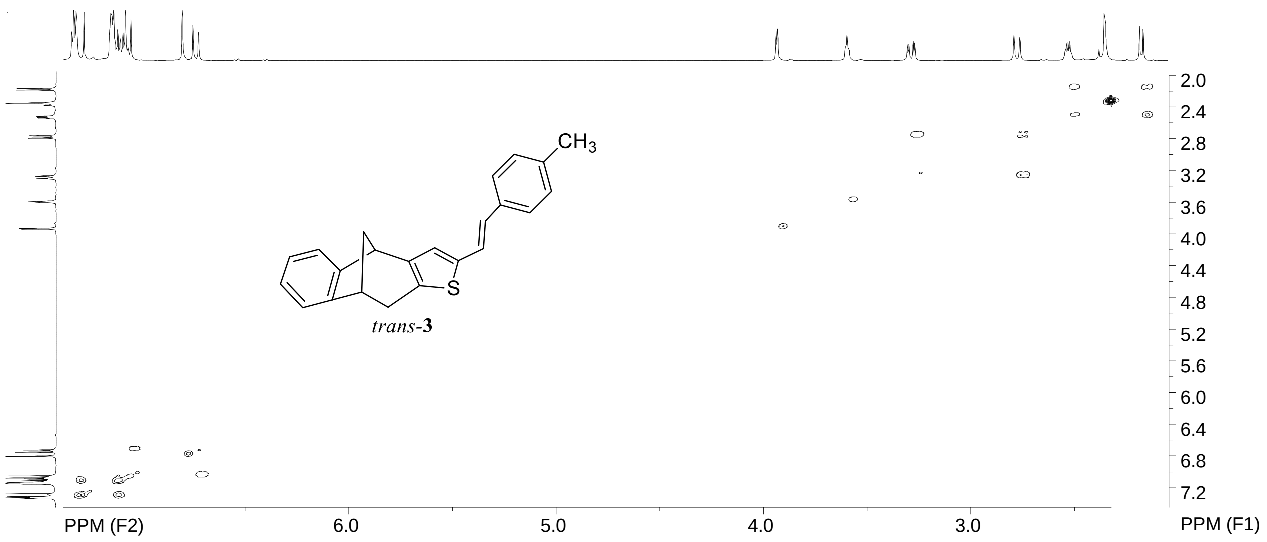

Figure S14: COSY spectrum ($CDCl_3$, 600 MHz) of compound *trans*-**3**

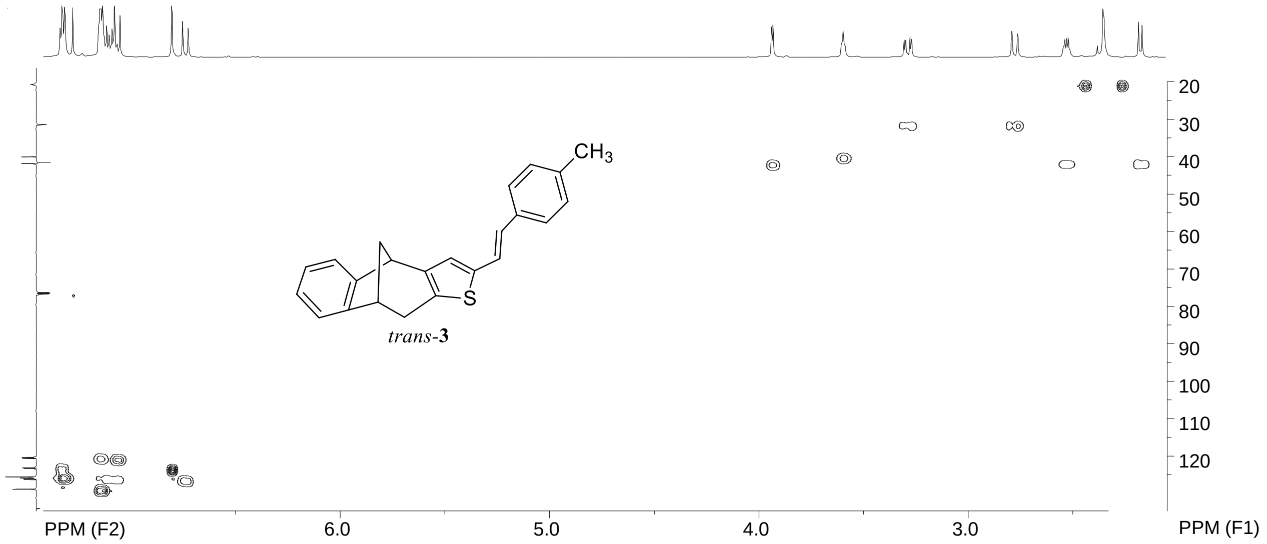

Figure S15: HETCOR spectrum ($CDCl_3$, 600 MHz) of compound *trans*-**3**

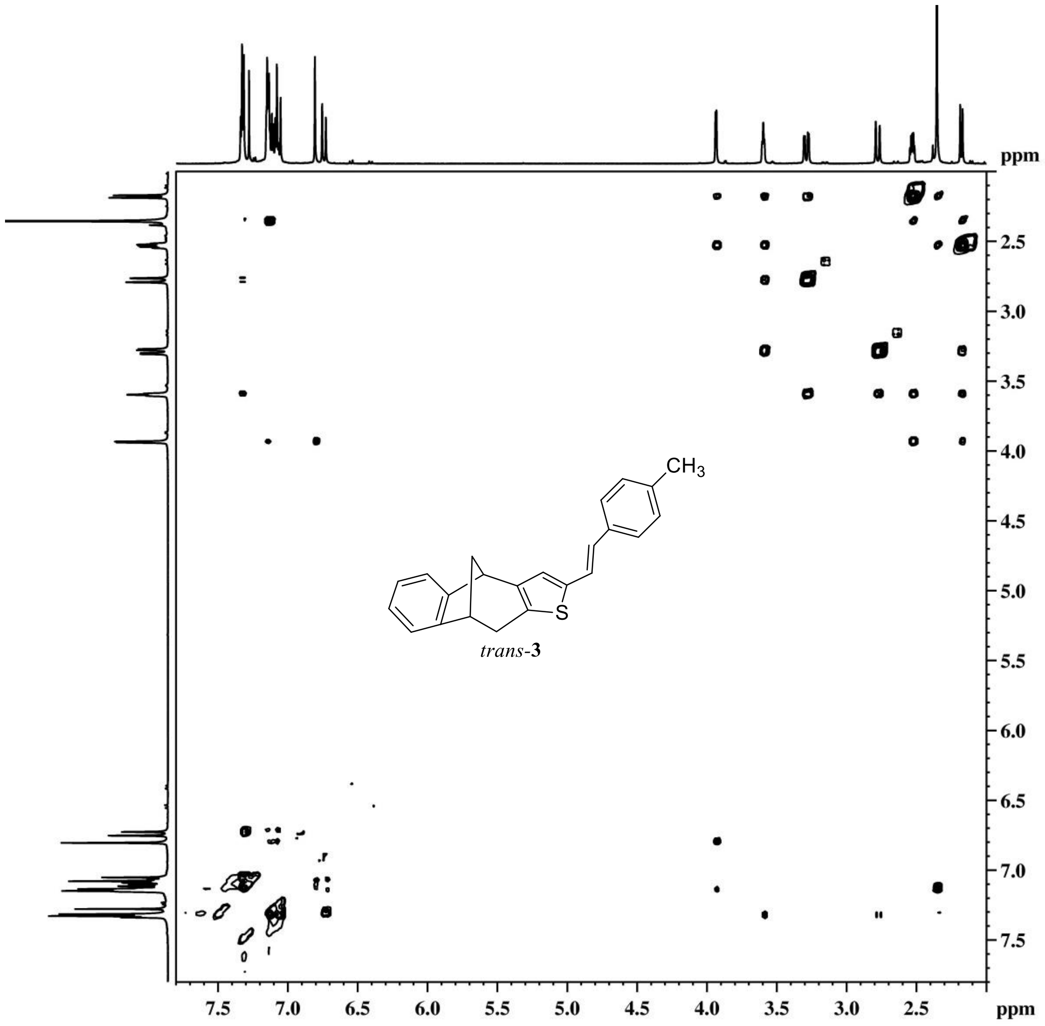

Figure S16: NOESY spectrum ($CDCl_3$, 600 MHz) of compound *trans*-**3**

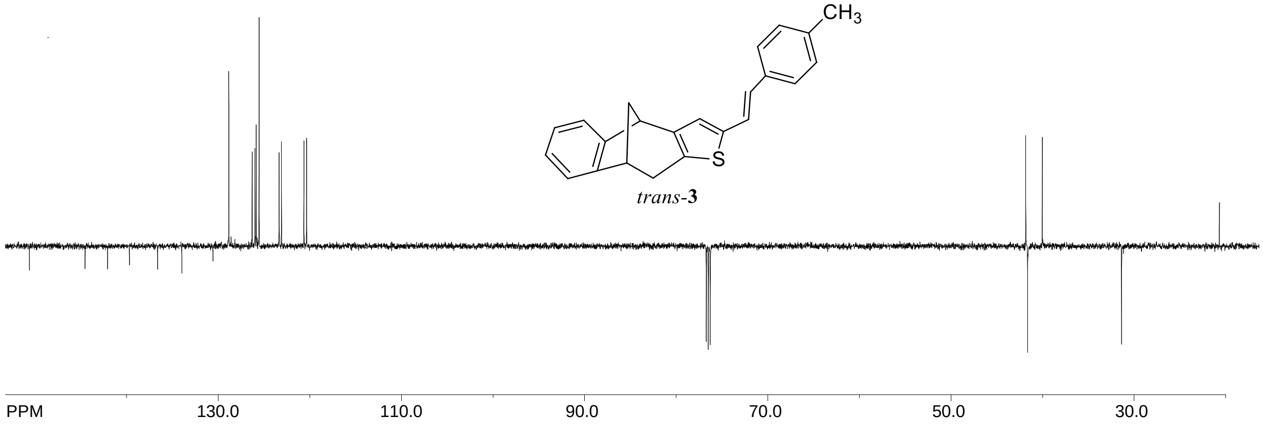

Figure S17: ^{13}C NMR spectrum ($CDCl_3$, 150 MHz) of compound *trans*-**3**

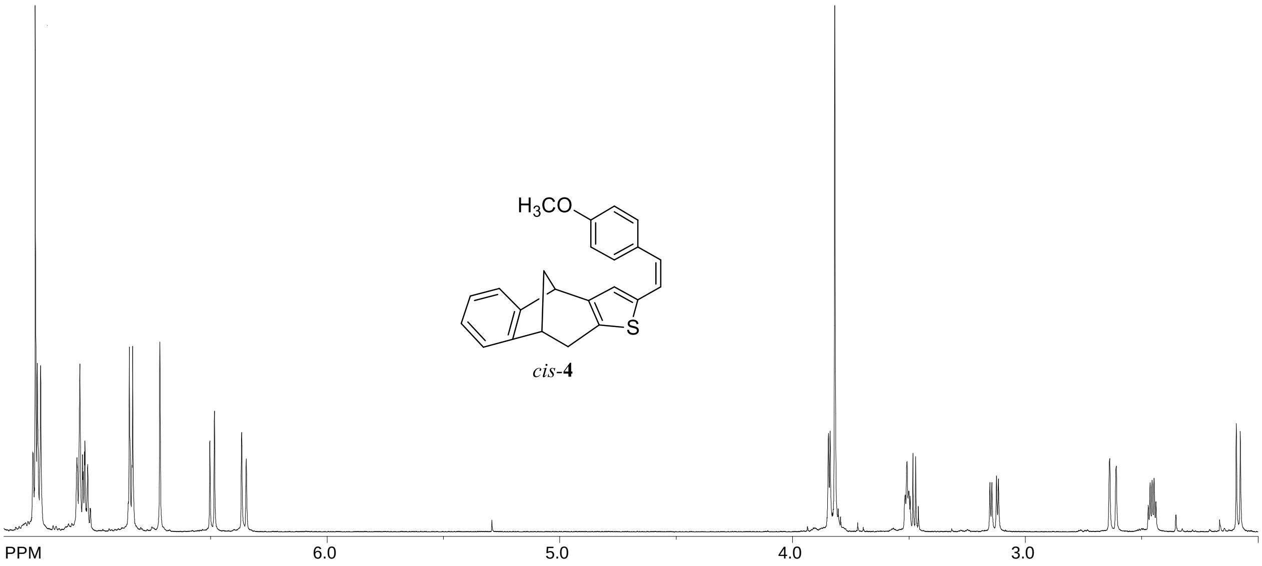

Figure S18: ^{1}H NMR spectrum ($CDCl_3$, 600 MHz) of compound *cis*-**4**

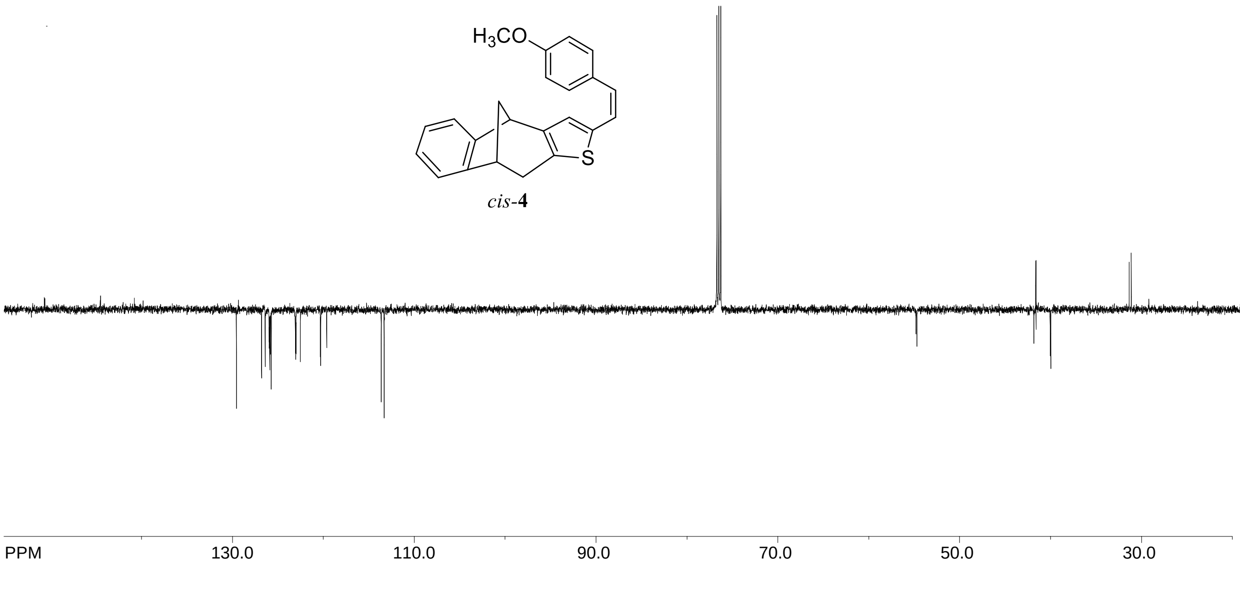

Figure S19: ^{13}C NMR spectrum ($CDCl_3$, 150 MHz) of compound *cis*-**4**

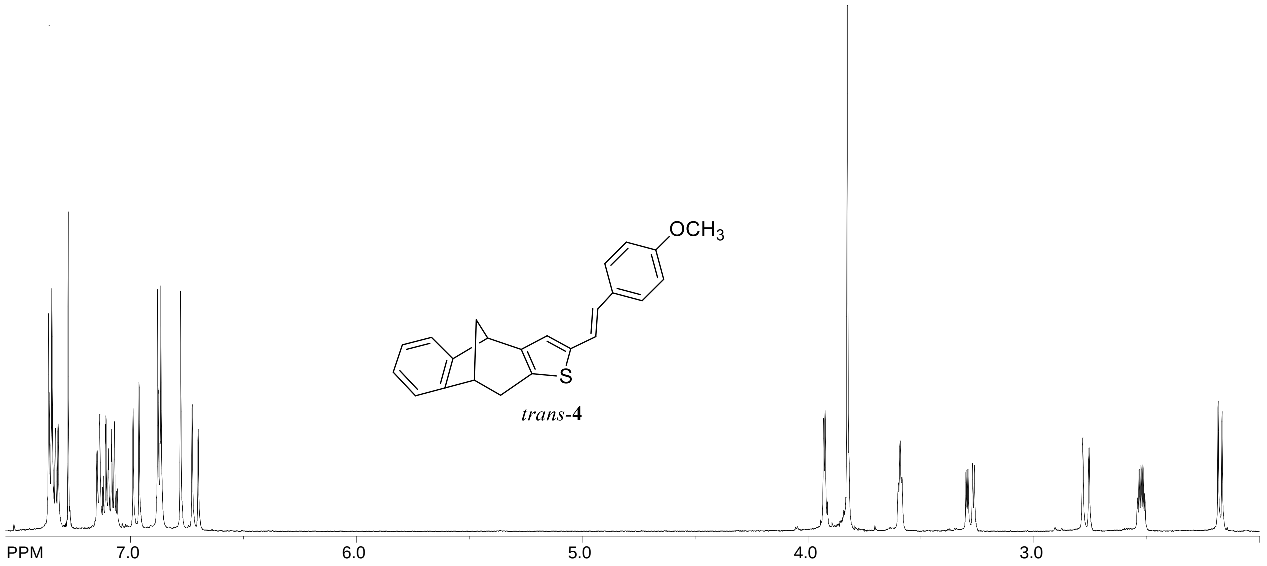

Figure S20: ^{1}H NMR spectrum ($CDCl_3$, 600 MHz) of compound *trans*-**4**

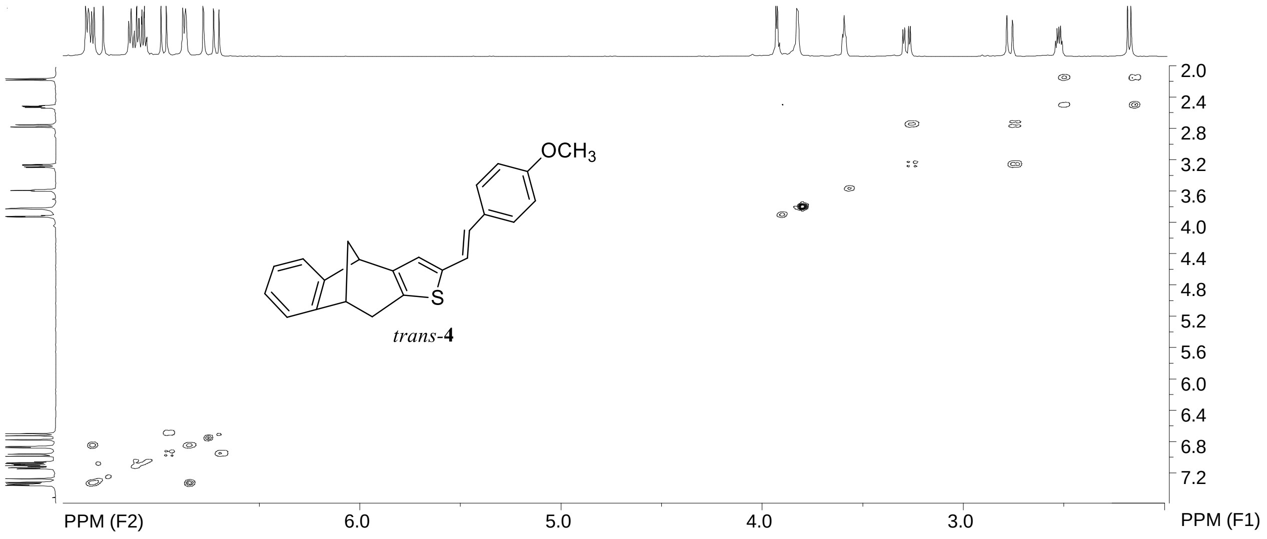

Figure S21: COSY spectrum ($CDCl_3$, 600 MHz) of compound *trans*-**4**

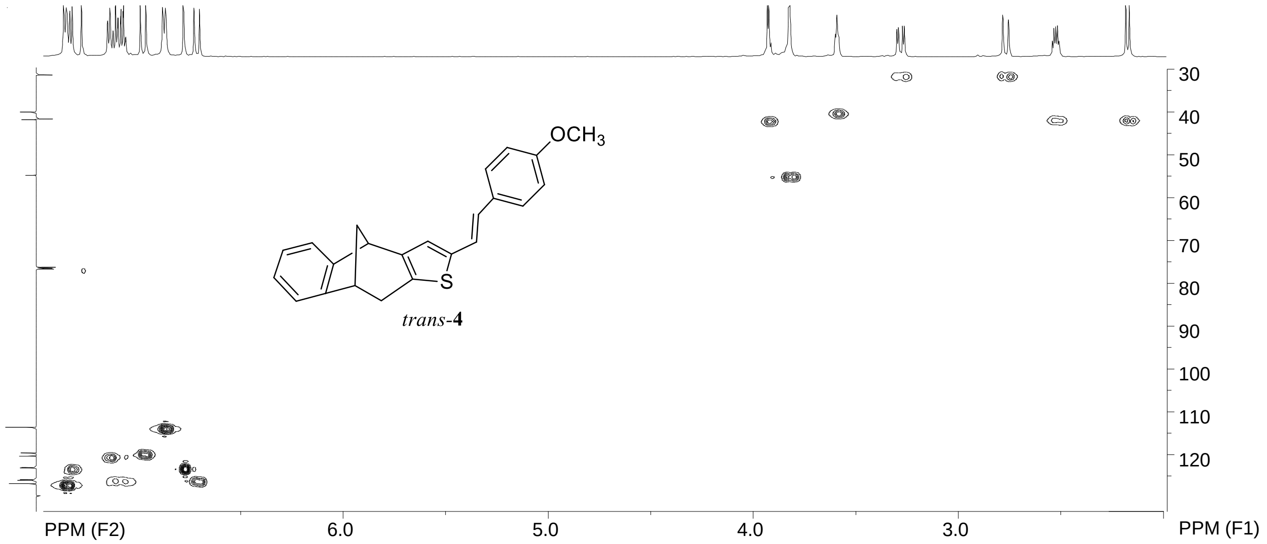

Figure S22: HETCOR spectrum ($CDCl_3$, 600 MHz) of compound *trans*-**4**

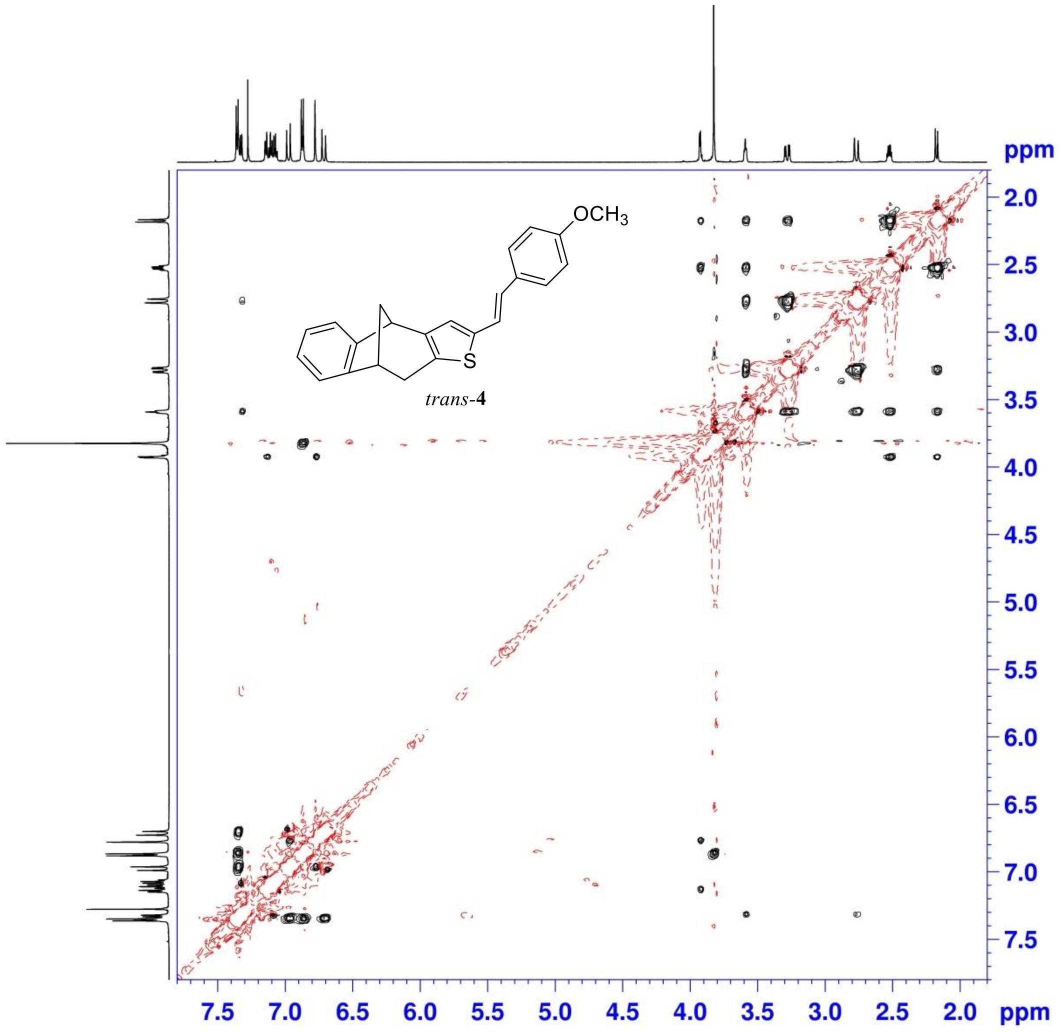

Figure S23: NOESY spectrum ($CDCl_3$, 600 MHz) of compound *trans*-**4**

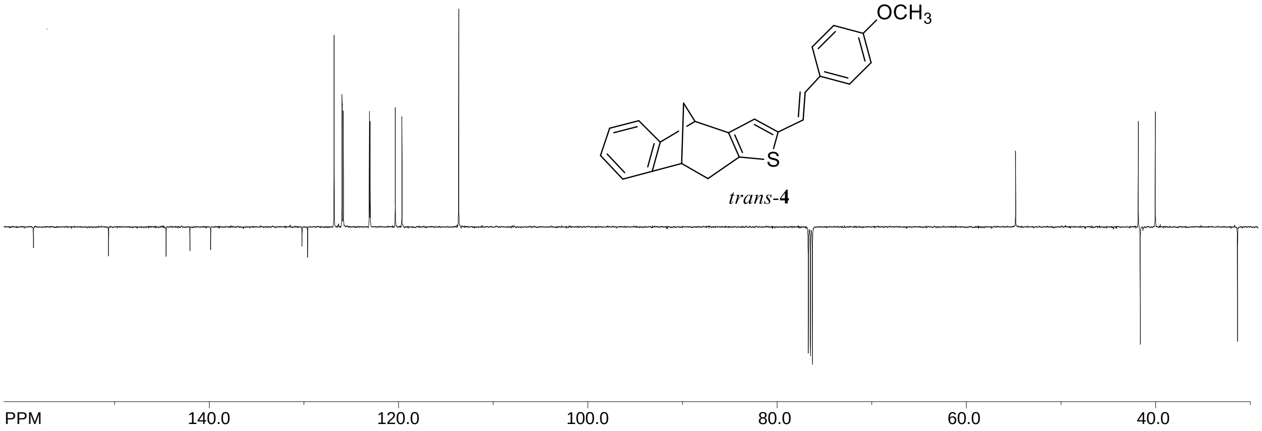

Figure S24: ^{13}C NMR spectrum ($CDCl_3$, 150 MHz) of compound *trans*-**4**

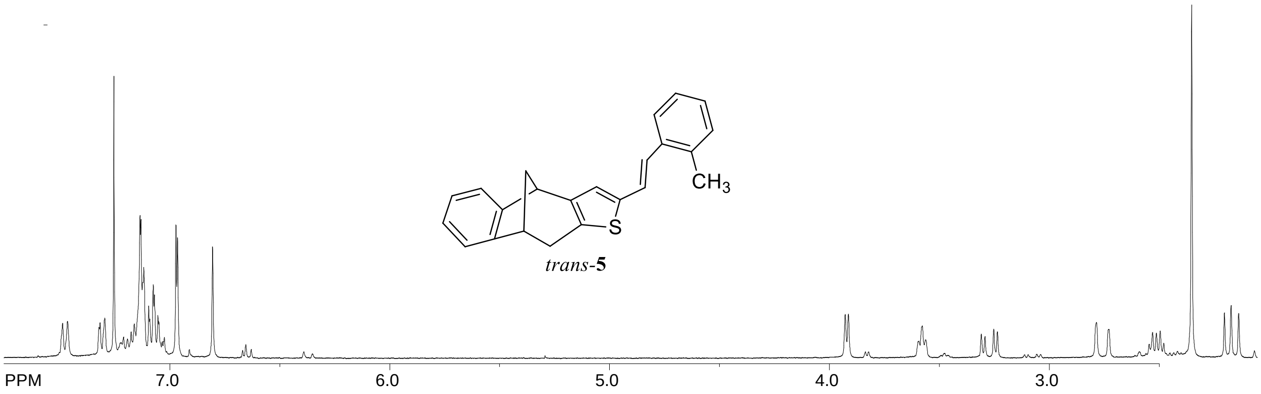

Figure S25: ^{1}H NMR spectrum ($CDCl_3$, 300 MHz) of compound *trans*-**5**

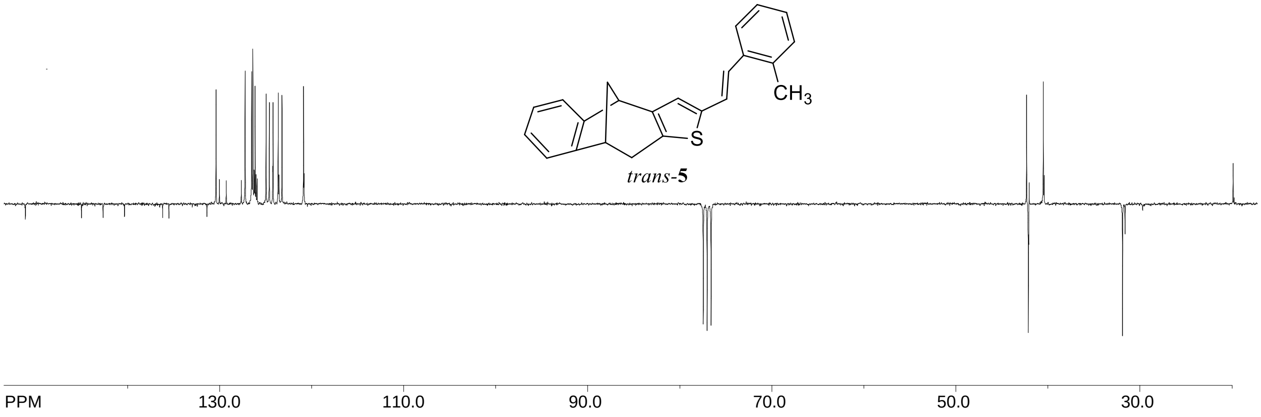

Figure S26: ^{13}C NMR spectrum ($CDCl_3$, 75 MHz) of compound *trans*-**5**

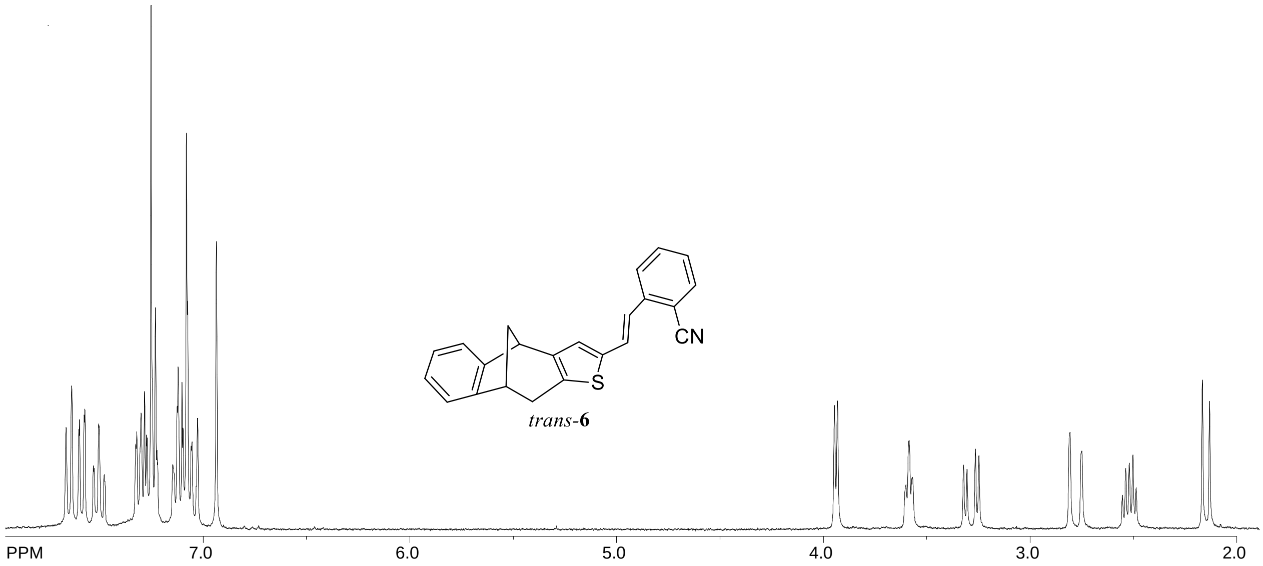

Figure S27: ^{1}H NMR spectrum ($CDCl_3$, 300 MHz) of compound *trans*-**6**

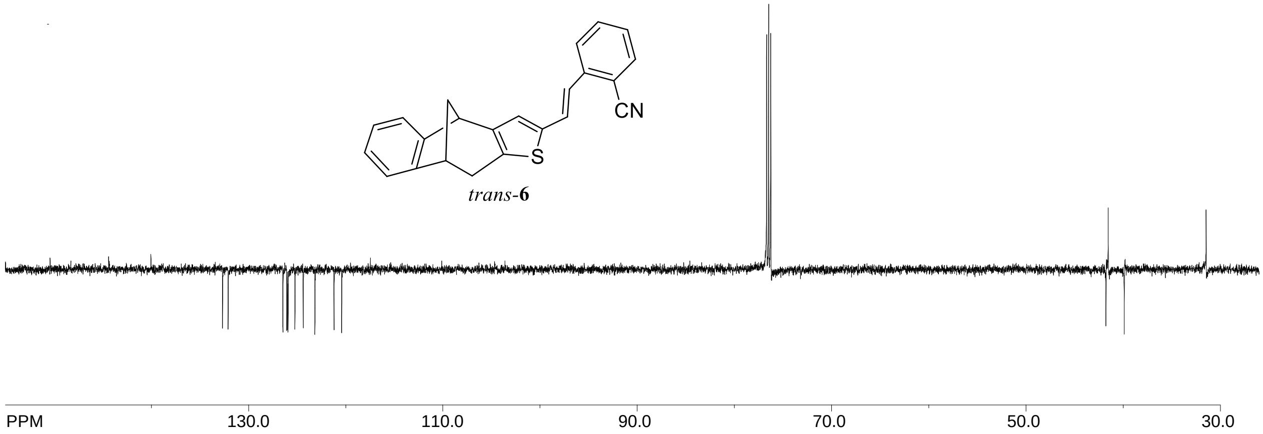

Figure S28: ^{13}C NMR spectrum ($CDCl_3$, 150 MHz) of compound *trans*-**6**

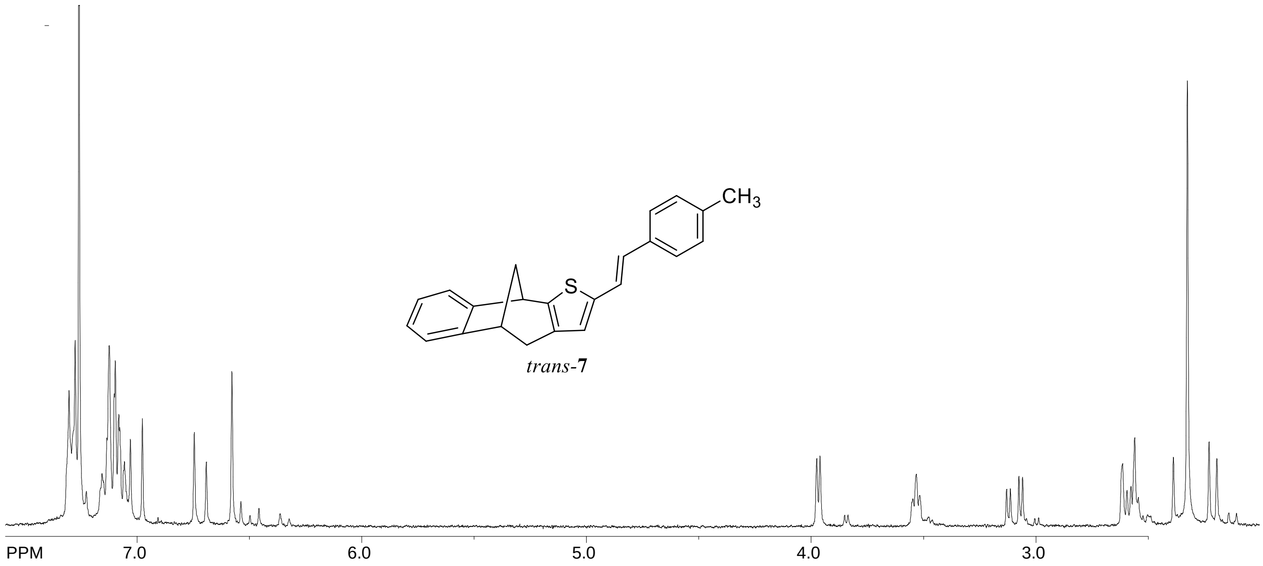

Figure S29: ^{1}H NMR spectrum ($CDCl_3$, 300 MHz) of compound *trans*-**7**

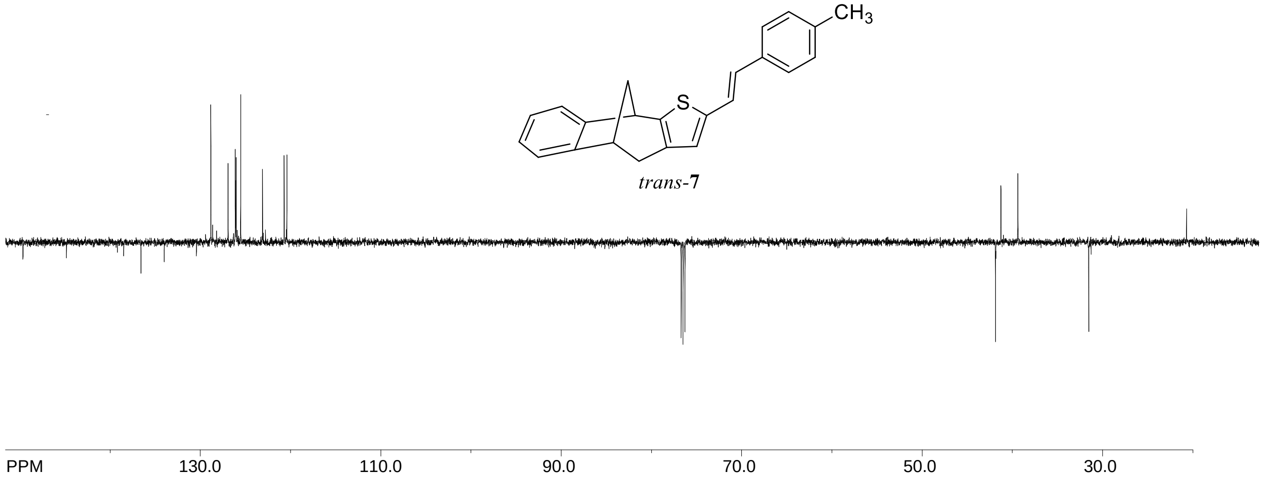

Figure S30: ^{13}C NMR spectrum ($CDCl_3$, 150 MHz) of compound *trans*-**7**

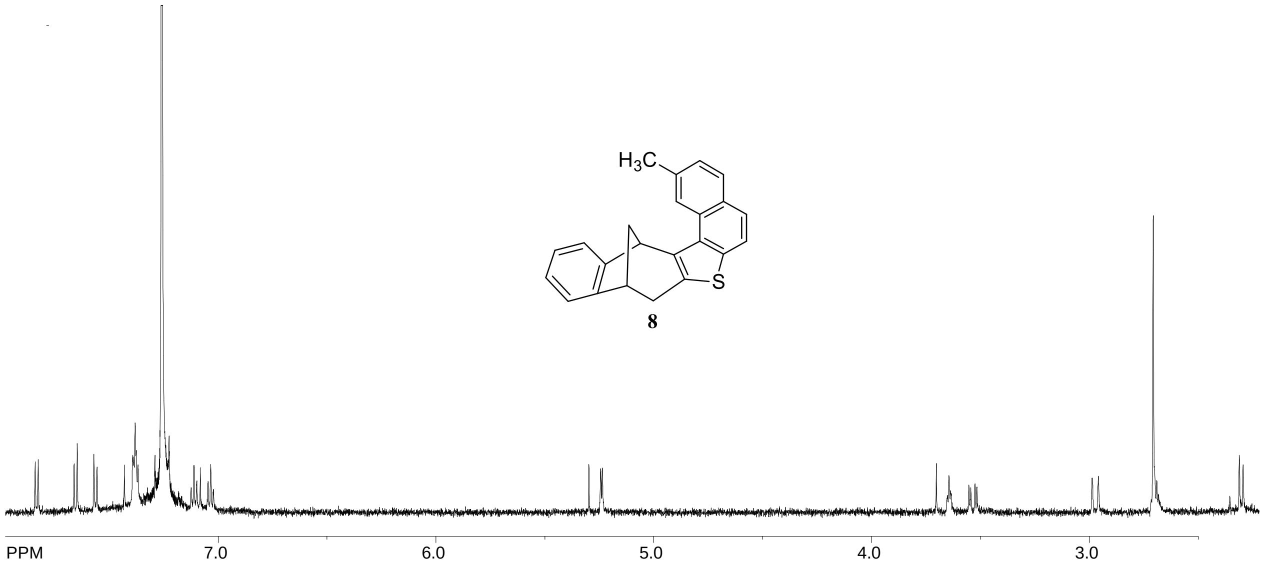

Figure S31: ^{1}H NMR spectrum ($CDCl_3$, 600 MHz) of compound **8**

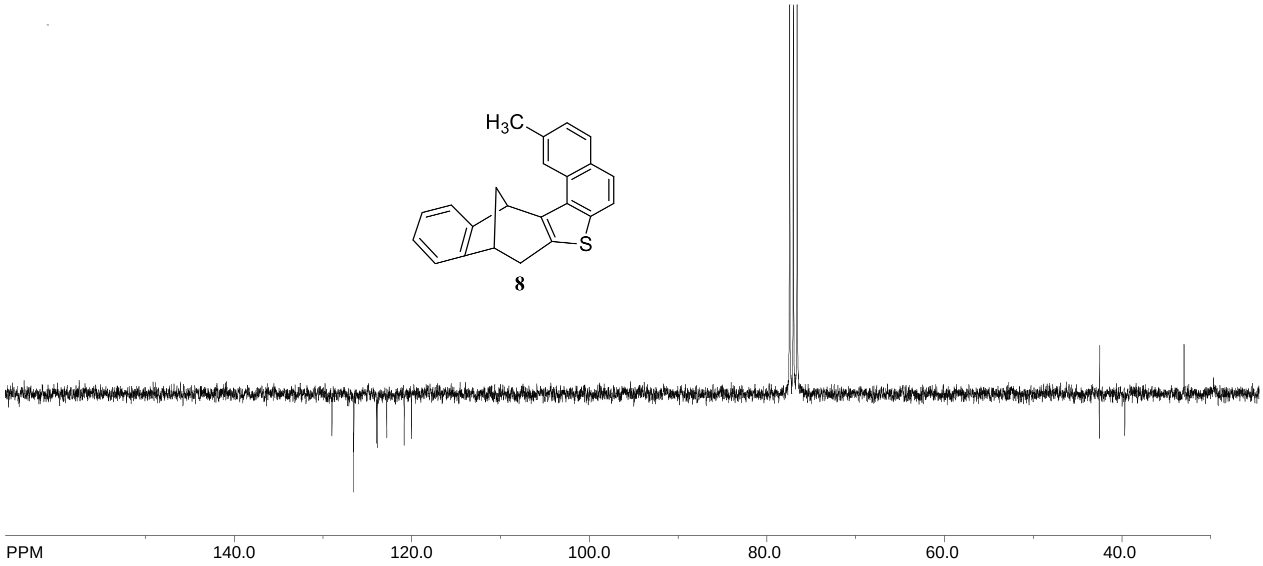

Figure S32: ^{13}C NMR spectrum ($CDCl_3$, 75 MHz) of compound **8**

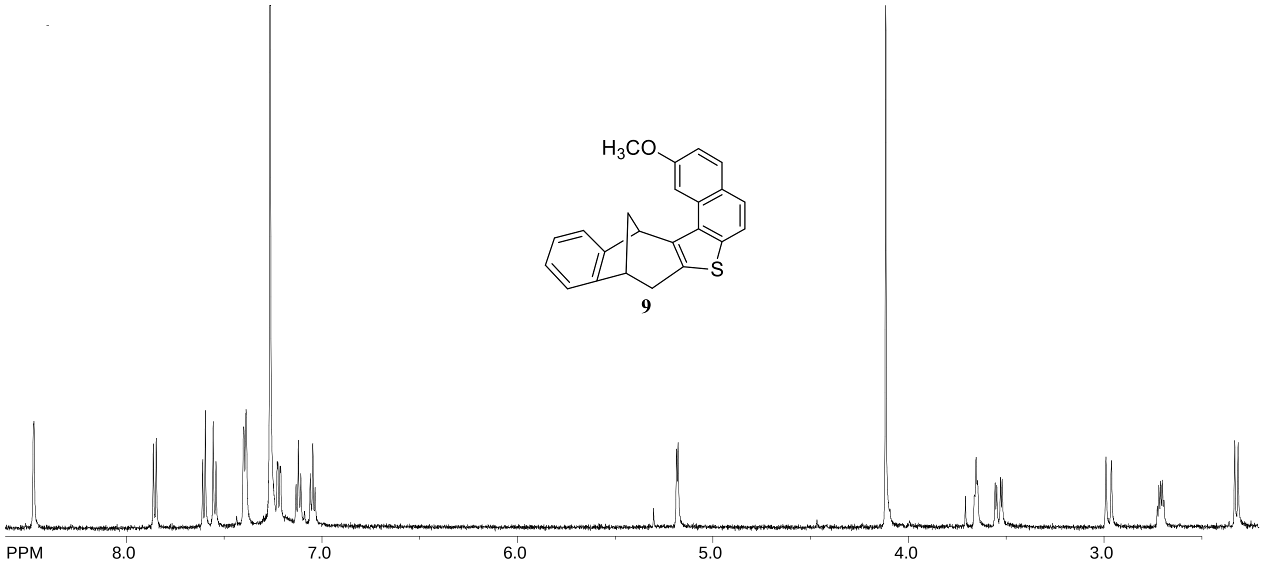

Figure S33: ^{1}H NMR spectrum ($CDCl_3$, 600 MHz) of compound **9**

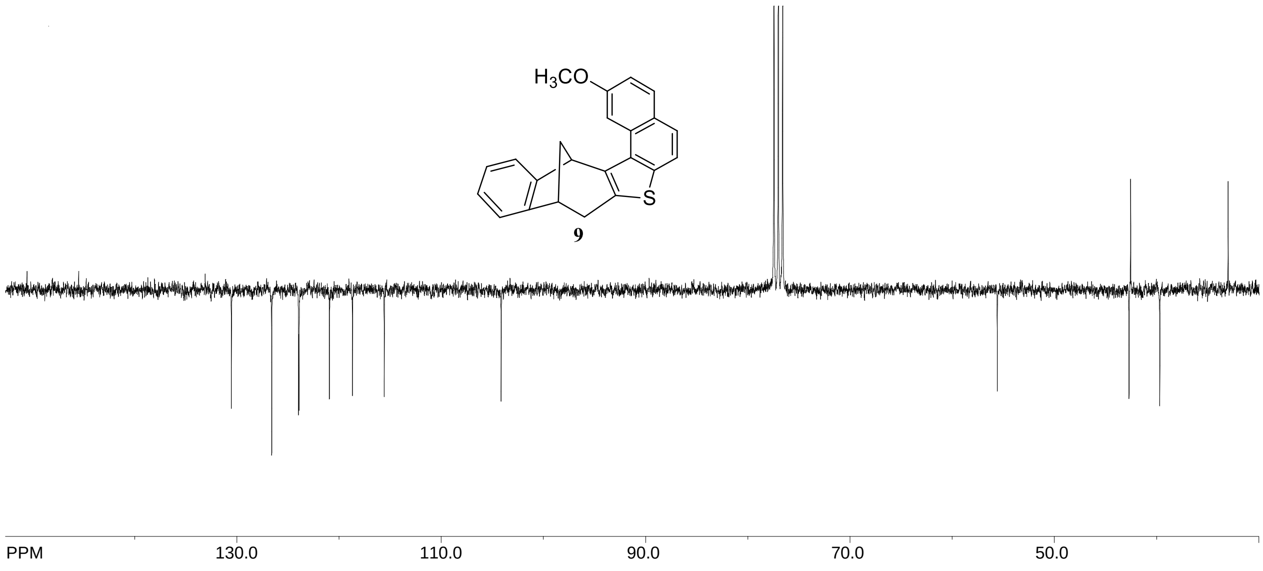

Figure S34: ^{13}C NMR spectrum ($CDCl_3$, 75 MHz) of compound **9**

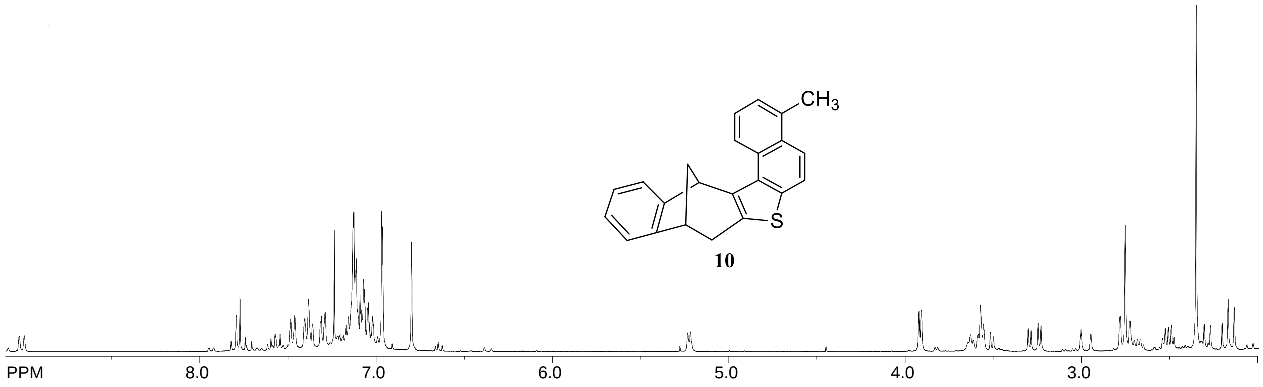

Figure S35: ^{1}H NMR spectrum ($CDCl_3$, 300 MHz) of compound **10**

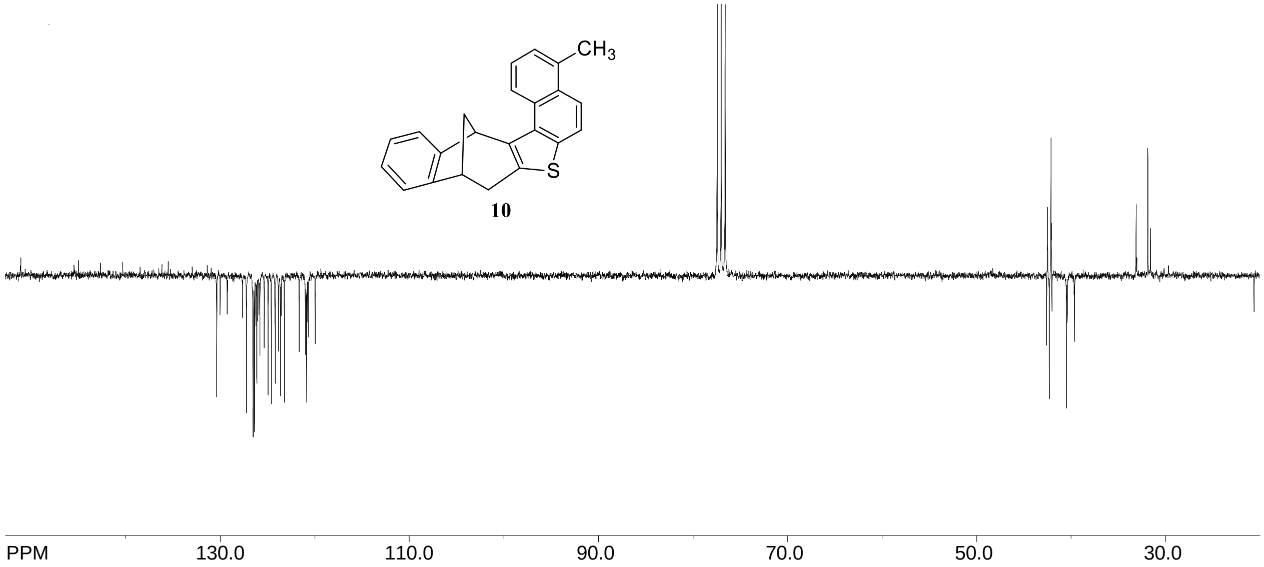

Figure S36: ^{13}C NMR spectrum ($CDCl_3$, 75 MHz) of compound **10**

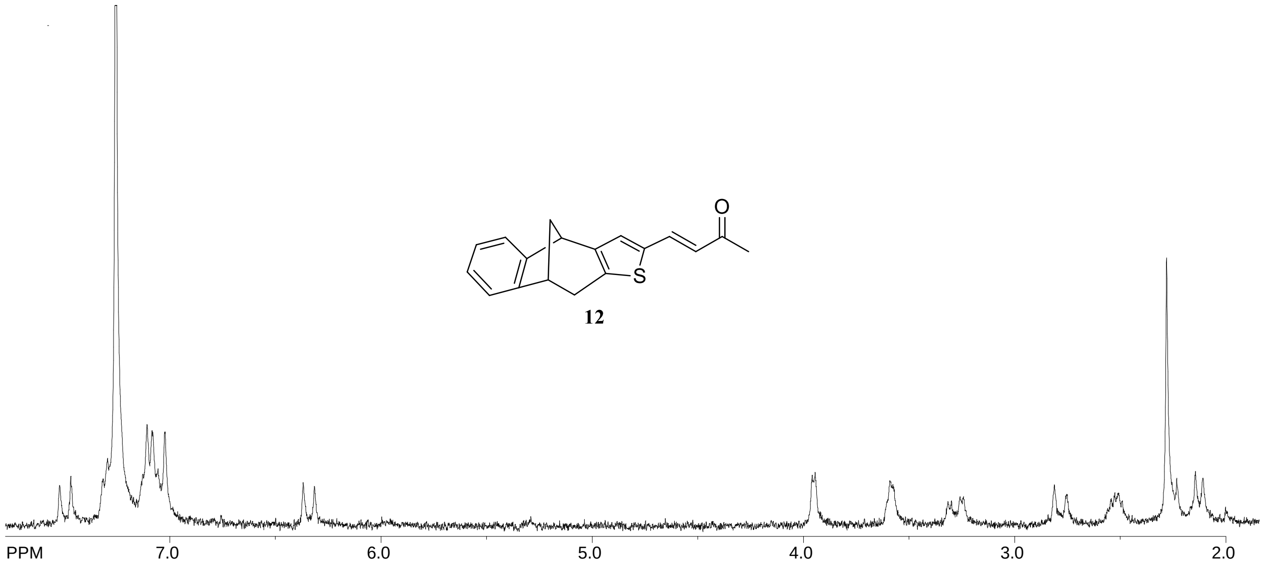

Figure S37: ^{1}H NMR spectrum ($CDCl_3$, 300 MHz) of compound **12**

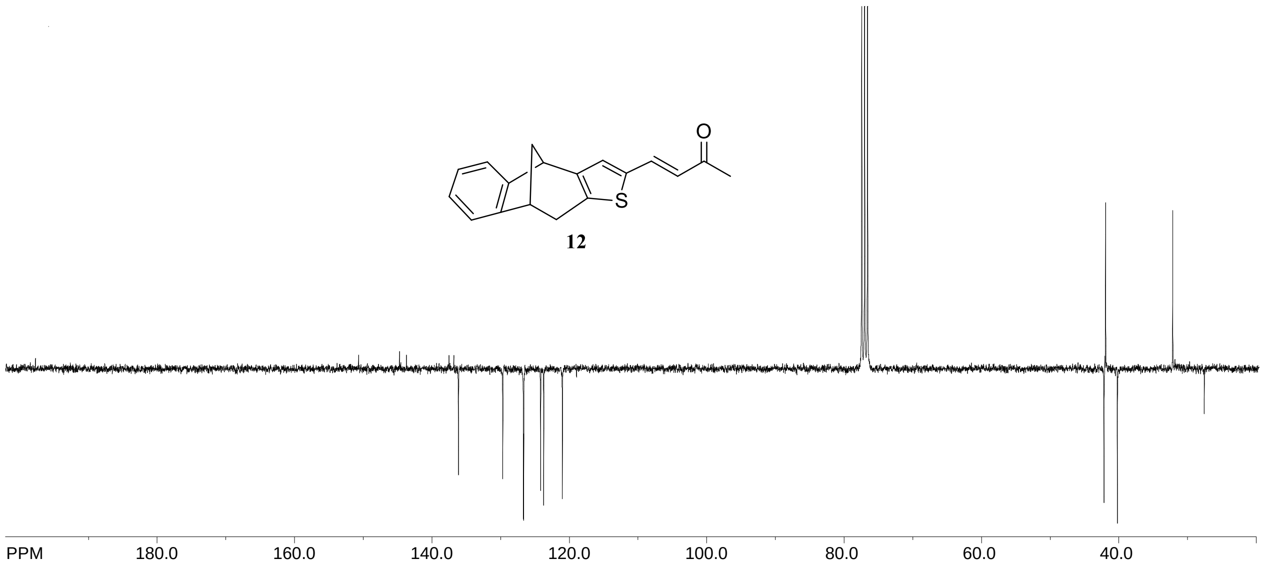

Figure S38: ^{13}C NMR spectrum ($CDCl_3$, 75 MHz) of compound **12**
